# *Toxoplasma gondii* drives myeloid immune cell recruitment to amyloid plaques in Alzheimer’s model mice

**DOI:** 10.1186/s12974-025-03666-2

**Published:** 2026-01-17

**Authors:** Katherine J. Olivia Yanes, Christina T. Bui, Julia Tomasello, Heba Morsy, Emilie Kim, Toan Lam, Kate Tsourmas, L. Angel Ayala, Kim N. Green, Matthew A. Inlay, Melissa B. Lodoen

**Affiliations:** 1 Department of Molecular Biology and Biochemistry, University of California Irvine, Irvine, CA 92697 USA; 2 Department of Neurobiology and Behavior, University of California Irvine, Irvine, CA 92697 USA; 3 Sue and Bill Gross Stem Cell Research Center, University of California Irvine, Irvine, CA 92697 USA

**Keywords:** 5xFAD, Myeloid cells, Microglia, Amyloid beta, Alzheimer’s disease, Monocytes, *T. gondii*

## Abstract

Infections in the central nervous system result in immune cell trafficking into the brain and microglial activation, which may influence Alzheimer’s Disease neuropathology. *Toxoplasma gondii* infection induces a robust neuroimmune response and a reduction in amyloid plaques in the brains of Alzheimer’s model mice. We investigated the myeloid cell response in the immediate vicinity of amyloid plaques in the brain by injecting 3-month-old 5xFAD mice with *T. gondii* or PBS as a control. *T. gondii* chronic infection (6 weeks) resulted in reduced amyloid plaque area, volume, and intensity in the cortex, and plaques with decreased circularity based on 6E10 and Thio-S staining. The brains of *T. gondii*-infected mice also had increased *AIF1*,* AXL*, and *CLEC7A* transcripts for disease-associated microglia (DAM), and elevated IBA1, MAC2, and CD68 phagolysosomal colocalization with amyloid, indicating myeloid cell activation around plaques. CD4 and CD8 T cells were also increased near amyloid and IBA1^+^ cells in *T. gondii-*infected mice. To determine the extent of peripheral myeloid cell recruitment to amyloid, bone marrow from CAG-CFP mice was transplanted into irradiated, head-shielded 5xFAD mice prior to infection. Cyan^+^ cells were found surrounding plaques in the brains of *T. gondii-*infected mice and were comprised predominantly of Ly6C^lo^ patrolling monocytes, followed by Ly6C^hi^ inflammatory monocytes and T cells. In addition, the majority of myeloid cells and T cells recruited to the brain were derived from skull bone marrow. These data demonstrate that *T. gondii* infection increases the infiltration of monocytes and T cells from the skull bone marrow niche and the recruitment of highly activated myeloid cells surrounding amyloid plaques in the brains of 5xFAD mice.

**Supplementary Information:**

The online version contains supplementary material available at 10.1186/s12974-025-03666-2.

## Background

Alzheimer’s disease (AD) is a neurodegenerative disease with characteristic neuropathology that includes the appearance of extracellular amyloid beta plaques and intracellular phosphorylated tau tangles. Neuronal damage also appears in the brains of AD patients, and the amyloid hypothesis ascribes this damage to the buildup of amyloid [1]. The appearance of neurological signs is often accompanied by a neuroinflammatory response, with microglia and other myeloid cells responding to the presence of amyloid plaques in the brain [2]. Genome-wide association studies in human Alzheimer’s patients, conducted to uncover potential risk and protective factors for AD, have revealed several altered genes expressed within myeloid cell populations and particularly in microglia. These include *TREM2*,* PLCG2*, and *ABI3*, reinforcing the potential importance of microglia in the disease process [3].

In the healthy brain, microglia are the resident myeloid cells, and they play important roles in debris removal and facilitating memory storage via synaptic pruning. Microglia are ontogenically distinct from other myeloid cells, as they are derived from the yolk sac [4, 5]. Once differentiated in the brain, microglia take on a homeostatic profile with outstretched processes and a specific transcriptional signature defined by the expression of *P2Ry12*,* TMEM119* and *CX3CR1* [5, 6]. However, in the context of Alzheimer’s disease, microglia display several distinct morphologies (ramified, ameboid, rod-shaped, dystrophic) and adopt an altered transcriptional program. In AD mouse models, microglial have been described as TREM2-independent or -dependent Disease Associated Microglia (DAMs), neurodegenerative microglia (MGnD), and activated response microglia (ARM) [5]. All of these transcriptional programs share an “activated” microglia phenotype, with high levels of *ITGAX* (CD11c), *CLEC7a*, and *Axl* [5, 7]. In the context of AD, monocytes may also be recruited to the brain in low numbers, and interestingly, appear to change their transcriptional profile to match that of “activated” microglia [5, 8]. Functionally, activated microglia and recruited monocytes increase phagocytosis of amyloid beta and have been suggested to play an important role in amyloid plaque dynamics [7, 9].

Infections in the CNS may also influence AD neuropathology through their activation of microglia and recruitment of immune cells. For instance, Alzheimer’s model mice infected with HSV-1 have elevated levels of amyloid beta, and when treated with PLX3397 to deplete microglia, have even higher amyloid load in the brain, suggesting that the microglial response to HSV-1 is important for limiting amyloid burden [10]. HSV-1 has also been suggested to be a potential causative agent of Alzheimer’s disease in APOE4 carriers [11, 12]. Conversely, infection of Alzheimer’s disease mice with the obligate intracellular parasite *Toxoplasma gondii* results in a decrease in amyloid plaques [13], which has been attributed to monocyte and microglia phagocytosis and removal of amyloid beta [14, 15]. Myeloid cells increase in the brains of AD mice during infection due to the infiltration of Ly6C^+^ monocytes and to proliferating microglia [13–15]. Monocytes isolated from the brains of *T. gondii-*infected AD mice are also more phagocytic, and depleting these cells reduces the effect of infection on amyloid plaque burden [14].

In this study, we sought to determine the dynamics of the interaction between myeloid cells, including both microglia and monocytes, and amyloid beta over the course of *T. gondii* infection as well as to determine the bone marrow source of these infiltrating myeloid cells. By infecting 5xFAD mice with *T. gondii* and analyzing brain sections by confocal microscopy, we observed increased colocalization of amyloid with myeloid cells and CD68 (a phagocytic marker) in infected mice. Bone marrow chimera experiments in 5xFAD mice demonstrated an increase in the recruitment of peripheral monocytes and T cells into the brain and near plaques due to infection, and these cells were derived from peripheral and skull bone marrow niches. Our data contribute to a growing picture of how immune cell infiltration into the Alzheimer’s brain may lead to amyloid reduction following infection.

## Methods

### Mice and infection experiments

All mouse husbandry and experimentation were approved by the University of California, Irvine Institutional Animal Care and Use Committee. The transgenic mouse strain used for this research project, B6.Cg-Tg (APPSwFlLon, PSEN1*M146L*L286V) 6799Vas/Mmjax, RRID: MMRRC_034848-JAX (5xFAD), was obtained from the Mutant Mouse Resource and Research Center (MMRRC) at The Jackson Laboratory, an NIH-funded strain repository, and was donated to the MMRRC by Robert Vassar, Ph.D., Northwestern University [16]. 5xFAD hemizygous and C57BL/6 mice were purchased from the NIH MMRRC or Jackson Laboratories, respectively, and bred in-house. CAG-CFP mice (Rosa-ECFP) were a generous gift from Dr. Irving Weissman [17]. All mice were infected at 3 months of age (12 weeks) with 200 GFP-expressing *Prugniaud* (type II, PA7 clone) tachyzoites in 200 µl of PBS injected intraperitoneally (i.p.). Control mice were injected i.p. with 200 µl of PBS. Both male and female mice were used in all experiments. Parasites were serially passaged in human foreskin fibroblasts (HFFs), as previously described [18] and harvested when they lysed out of HFFs. Mycoplasma testing was completed for all parasite and cell lines bimonthly and confirmed to be negative. At necropsy, mice were injected with 2.5% Tribromoethanol (Avertin, Sigma-Aldrich), blood was collected via cardiac puncture, and mice were transcardially perfused with 50 mL of 1x PBS (Corning) to remove non-adherent cells in the vasculature. The brains were harvested from the mice, and individual brain hemispheres were further processed as described below.

### Gene expression analysis by qPCR

Intact brain hemispheres from infected and uninfected 5xFAD mice were collected in RNAlater. Tissues were then minced and homogenized, and 30 mg of tissue was processed using a RNeasy kit (Qiagen, Germantown, MD). cDNA was generated, and the following primers were used to amplify target genes: *clec7a* (forward, F) 5’ CCCAACTCGTTTCAAGTCAG 3’, (reverse, R) 5’ AGACCTCTGATCCATGAATCC 3’ [19]; *axl* (F) 5’ GGTGGTTGAGCCAACCGTGGA 3’, (R) 5’ GCCACCTTATGCCGATCTACCA 3’ [20]; and *aif1* (F) 5’ CCAGCCTAAGACAACCAGCGT 3’, (R) 5’ GCTGTATTTGGGATCATCGAGGAA 3’ [21]. Mouse glyceraldehyde-3-phosphate dehydrogenase (*gapdh*) was amplified for comparison with the following primers: (F) 5’ GCATGGCCTTCCGTGTTC 3’, (R) 5’ GATGTCATCATACTTGGCAGGTTT 3’ [22]. qPCR was performed in triplicate with SYBR green Supermix (Bio-Rad, Hercules, CA) on the Bio-Rad iCycler. Expression was quantified using the threshold cycle method, followed by normalization to the levels of *GAPDH* [23].

### Immunofluorescence staining and imaging

After harvest, one hemisphere of the brain was incubated in 4% PFA overnight, followed by incubation in 30% sucrose in PBS until the tissue sank, and then embedded in OCT freezing medium (Thermo Fisher Scientific) for cryopreservation. Brains were sectioned using a cryoslicer to 16 μm sections and directly placed onto glass slides. For imaging amyloid in all animals other than irradiated animals, sections were rinsed in PBS for 15 min at room temperature (RT), stained for amyloid using Amylo-Glo (TR-200-AG; Biosensis, Thebarton, South Australia) at 1:100, then blocked and permeabilized in IFA buffer [1x PBS, 3% normal goat or donkey serum (Southern Biotech, Birmingham, AL), 0.3% Triton-x 100 (Thermo Fisher Scientific)]. For imaging amyloid in irradiated animals, Amylo-Glo was used at 1:1000 to reduce spectral overlap between unbound Amylo-Glo and cyan fluorescent protein [24]. The following antibodies were used to stain tissue sections overnight in IFA buffer: mouse anti-amyloid 6E10 (Ultra-LEAF Purified anti-b-Amyloid, 1–16, clone 6E10, Biolegend) at 1:1000, followed by donkey anti-mouse AlexaFluor 647 (Invitrogen); rabbit anti-IBA1 (Fujifilm Wako, Japan) at 1:1000, followed by goat anti-rabbit AlexaFluor 647 or AlexaFluor 594 (Invitrogen) at 1:500; rat anti-CD68 (clone FA-11, Bio-Rad) at 1:500, followed by goat anti-rat AlexaFluor 594 at 1:500; rat anti-mouse/human MAC-2 (Galectin-3) (Cedarlane Laboratories, Burlington, NC), followed by goat anti-rat AlexaFluor 647 at 1:500; goat anti-MAC2 (1:300), followed by donkey anti-goat AlexaFluor 647 (1:500); goat anti-IBA1, followed by donkey anti-goat AlexaFluor 594 (Invitrogen). For Thio-S and 6E10 staining, sagittal brain sections were immersed in citrate buffer at 80˚ C for 20 min for antigen retrieval, then blocked and permeabilized in IFA buffer [1x PBS, 3% normal donkey serum (Southern Biotech, Birmingham, AL), 0.3% Triton-x 100 (Thermo Fisher Scientific), 1:28 Mouse on Mouse Protein Concentrate (Vector Laboratories)]. After staining with the 6E10 antibody, amyloid was then labeled with filtered 0.5% w/v Thioflavin S (Sigma-Aldrich) in 50% EtOH for 10 min at RT. These sections were washed with a series of 50% EtOH, PBS, and water prior to mounting. For CD4 and CD8 staining, sagittal brain sections were immersed in citrate buffer at 80˚ C for 20 min for antigen retrieval, then blocked and permeabilized in IFA buffer [1x PBS, 3% normal donkey serum (Southern Biotech, Birmingham, AL), 0.3% Triton-x 100 (Thermo Fisher Scientific)], and incubated overnight with the following antibodies: rabbit anti-CD4 clone BLR167J (Invitrogen) (1:100) and rat anti-CD8a clone 4SM16 (eBioscience) (1:100), followed by donkey anti-rat AlexaFluor 647 (Jackson ImmunoResearch) (1:500) and donkey anti-rabbit AlexaFluor 594 (Jackson ImmunoResearch) (1:500).

Sections were imaged using a Leica Sp8 microscope with an oil immersion 40x objective. For confocal area analysis of Amylo-Glo, 2–4 z-stacks from 1 to 2 sections per animal were maximally projected then analyzed using FIJI to calculate the percent of amyloid area per field of view (FOV) and averaged per mouse. For area, intensity, and circularity of Thio-S and 6E10, ten z-stacks across the cortex from 2 sections per animal were taken. The FOVs were maximally projected, analyzed using FIJI, and averaged per mouse. Circularity, also called shape index, was quantified in FIJI using the standard equation: circularity= (4*pi*area)/(perimeter^2^) [25]. For quantification of IBA1^+^, CD68^+^ and MAC2^+^ signal and colocalization with Amylo-Glo, 2–6 FOVS in the cortex with one section per animal were quantified and averaged. IBA1^+^, Amylo-Glo, MAC2^+^, cyan^+^ signal and colocalization were quantified in 2–8 FOVs with cyan^+^ signal across the cortex from 2 sections per animal in PBS and *T. gondii*-infected chimeric mice and averaged per mouse. Imaris (Oxford Instruments) was used for confocal volume analyses of Amylo-Glo, IBA1, CD68, MAC2, CD4, CD8, amyloid and colocalization. Colocalization of IBA1 and CD68 was defined as any volume within the z-stack that was both CD68^+^ and IBA1^+^ (and did not include CD68^+^IBA1^−^ or CD68^−^IBA1^+^ signal). Cellular proximity to plaque cores was determined in Imaris using a distance threshold, as specified, and normalized to the total number of plaques per FOV. CD4^+^ and CD8^+^ T cells wrapped in IBA1 were manually quantified by an investigator blinded to the identity of the samples.

### Flow cytometry

Brain hemispheres were homogenized to a single cell suspension following manual mincing of brain tissue and dispase II (Toche Applied Science) digestion at 37˚ C in HEPES-buffered saline. The solution was further triturated and passed through a 70 μm filter (Falcon), and myelin was removed using a 35% and 75% percoll (GE Healthcare) gradient. Peripheral blood from chimeric mice was collected via the tail vein in K_2_EDTA (SAI)-coated tubes, and red blood cells were lysed with ACK lysis buffer (Life Technologies). The resulting white blood cells were resuspended in Hank’s Balanced Salt Solution (HBSS) with 5% FBS and filtered through a 70 μm filter. Brain and blood cells were resuspended in 10% TrueStain FcX (Biolegend) in staining buffer (3% FBS in PBS). Cells were then stained with directly conjugated antibodies from Biologend: CD45 BV785 (Cat: 103149), CD11b BV605 (Cat: 101257), Ly6G BV421 (Cat: 127628), Ly6C PerCP/Cyanine5.5 (Cat:128012), CD3 APC/Cyanine7 (Cat:100222), Zombie Yellow Dye in Fixable Viability Kit (Cat: 423103/423104), CD19 PE (Cat: 115508), CD11c PE/Cyanine7 (Cat: 117318), NK1.1 APC (Cat:108709). Cells were analyzed using a NovoCyte flow cytometer (Agilent), and the data were analyzed using FlowJo software (Treestar). All gating in the flow cytometry plots was based on fluorescence minus one (FMO) controls.

### Bone marrow chimeras

5–7 week-old 5xFAD and C57BL/6 mice were irradiated and reconstituted with CAG-CFP-expressing bone marrow. To isolate bone marrow, femur bones from C57BL/6 CAG-CFP mice were cut at the ends and centrifuged at 5000 rpm for 30 s. Cells were then resuspended in 1 mL of ACK buffer for red blood cell lysis, resuspended in HBSS with 5% FBS, centrifuged at 450 x g for 5 min, resuspended in PBS, then filtered through a 70 μm filter and counted. 5xFAD mice were anesthetized with 2% isoflurane in O_2_ and maintained at 1% isoflurane for the duration of radiation and retro-orbital injection. Anesthetized mice were head-shielded using a lead block and irradiated using a Precision X-Rad 320 Irradiator with 825 cGrey. The fur of the irradiated mice turned gray, except on their heads, which remained black, indicating effective head shielding. 2 × 10^6^ bone marrow cells in 100 µl of PBS from sex-matched C57BL/6 CAG-CFP mice were then retro-orbitally injected into the irradiated 5xFAD mice. Irradiated mice were observed for 15 min on a heat pad and kept on a UNIPRIM (antibiotic-containing) diet for 3 weeks, then returned to standard chow thereafter. Peripheral blood from the tail vein was collected from the mice at 11 weeks of age to determine chimerism. Chimerism was determined based on the percentage of granulocytes in the peripheral blood, since granulocytes are short-lived [26]. Chimeric mice were then infected at 12 weeks of age.

### Statistics

All statistical analysis was done in consultation with the Biostatistics, Epidemiology, and Research Design (BERD) unit of the Institute for Clinical and Translational Sciences (ICTS) at UC Irvine. Experimental outcomes were tested for significance using Student’s *t*-test and one- or two-way ANOVA (as appropriate) followed by post hoc testing (Sidak’s, Tukey’s or linear step procedure of Benjamini, Krieger, and Yekutieli). Graphs were generated in Prism. The number of samples per group and significance for each comparison are in each figure legend. Error bars in all figures represent standard deviations, unless otherwise stated.

## Results

### Chronic *T. gondii* infection reduces amyloid in cortical and hippocampal regions of 5xFAD mice

**Fig. 1:**
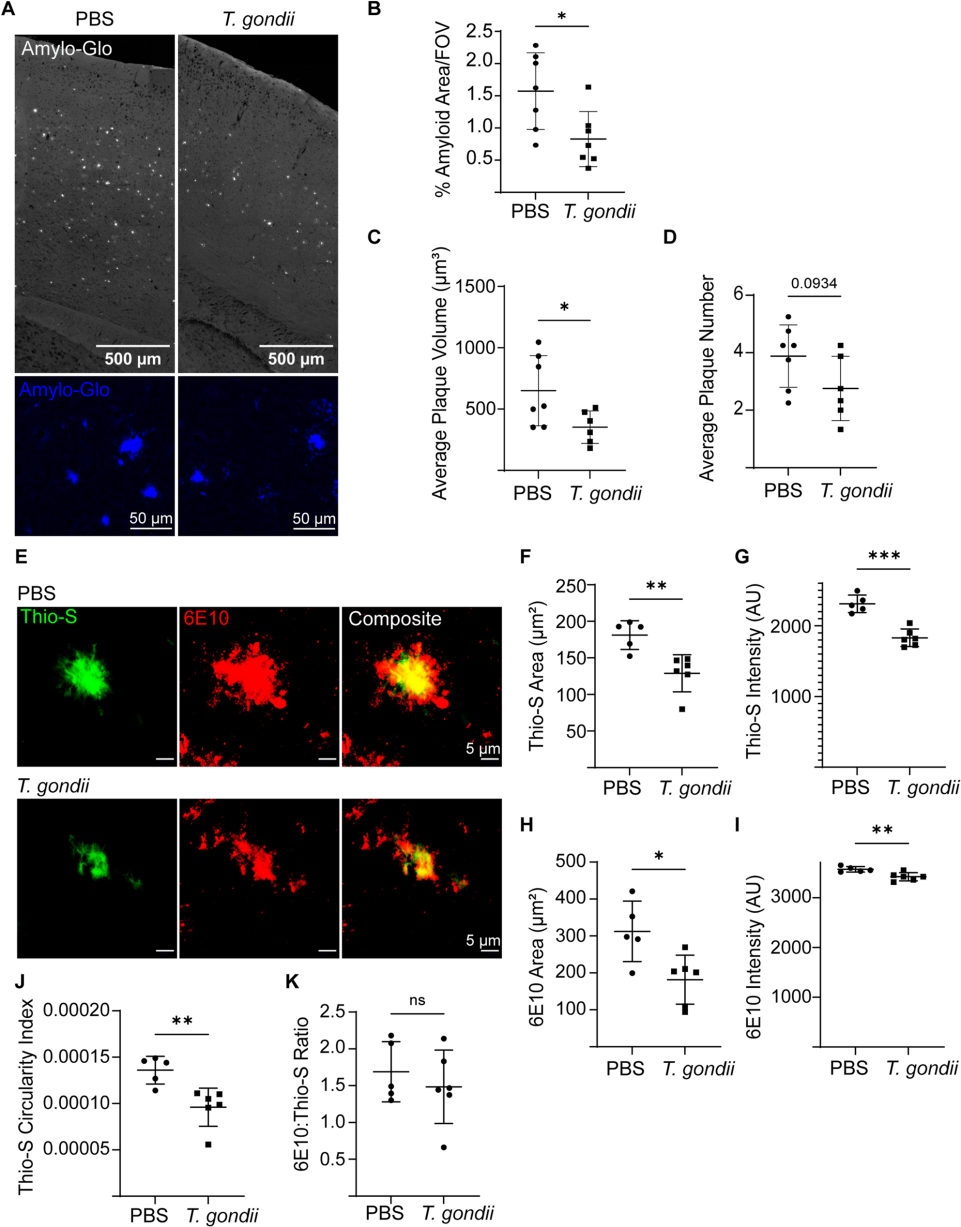
Amyloid burden and circularity are significantly reduced in the cortex of *T. gondii*-infected 5xFAD mice. Three-month-old 5xFAD mice were injected i.p. with PBS or type II *T. gondii*, and cortical amyloid was analyzed at 6 wpi. **A** Representative images show amyloid (Amylo-Glo) signal in the cortex of PBS or *T. gondii*-injected mice. **B** Average percent of amyloid area per FOV in each mouse. **C** Average volume of amyloid plaque cores per FOV. **D** Numbers of amyloid plaques per FOV. **E** Representative images of plaques in the cortex labeled with Thio-S (green) and 6E10 (red) in PBS and *T. gondii*-infected animals. Thio-S and 6E10 area (**F**, **H**) and intensity (**G**, **I**) were measured for each plaque and averaged in each mouse. **J** Thio-S circularity index in each mouse (where 1 is a perfect circle and 0 is elongated). **K** Quantification of 6E10: Thio-S area ratio averaged for each mouse. In B-D *n*=6-7 mice per group. In F-K, *n*=5-6 per group. In all graphs, each dot is one mouse. Student’s t-test, *p<0.05, **p<0.01, ***p<0.001, ns: not significant

We and others have observed that infection of AD model mice with *T. gondii* reduces amyloid plaque density [13–15, 27, 28]. However, a detailed characterization of plaque shape and composition in response to infection has not been reported. To address this, we injected PBS or type II *T. gondii* intraperitoneally (i.p.) into 3-month-old 5xFAD mice and examined amyloid plaques in high resolution in the brain at 6 weeks post-infection (wpi), which is 4.5 months of age. Cryopreserved brain sections were stained with Amylo-Glo, a styrylbenzene derivative to detect dense core amyloid and peripheral fibrils of amyloid, similar to Thioflavin-S [24, 29, 30]. Confocal microscopy was used to image cortical brain sections from control or *T. gondii-*infected 5xFAD mice (Fig. 1A). The percent area of amyloid signal and average volume of amyloid plaques in maximum z-projections from multiple fields of view (FOV) in the cortex were measured. Amyloid area and core volume were significantly decreased in the cortex of infected mice (Fig. 1B and C), and there was a trending decrease in the number of plaques (Fig. 1D). However, there was no statistically significant difference in the area, volume or plaque number in the subiculum following infection (Sup. Figure 1A-D).

Notably, the amyloid plaques in the cortex of *T. gondii*-infected animals were not only reduced in size, but also appeared more diffuse, with increased filamentous morphology and decreased fluorescence intensity, compared to those in the cortex of PBS-injected control 5xFAD mice. To quantify these differences, we labeled plaques with Thioflavin-S (Thio-S), a thioflavin dye that binds amyloid fibrils, particularly those in dense cores, and 6E10, an antibody that binds to the amyloid amino acid residues 4–10, labeling monomers and fibrils in diffuse plaques [31–33]. Confocal z-stacks of individual plaques in the cortex were acquired, and amyloid composition and morphology were analyzed from the maximal z-projections (Fig. 1E). Both Thio-S and 6E10 area and intensity, per plaque, were significantly decreased following infection (Fig. 1F-I). We also measured circularity, or shape index, which accounts for the relationship between area and perimeter (1 is a perfect circle and 0 represents an elongated or irregular shape). The circularity of the amyloid plaques labeled by Thio-S was significantly decreased following infection (Fig. 1J). Since both the Thio-S and 6E10 signals were reduced in infected mice, the ratio of the two, a common measurement used as a proxy for diffuseness of amyloid plaques, was not significantly changed following infection (Fig. 1K). Altogether, this analysis of amyloid morphology suggests that amyloid plaques are both smaller and less circular in *T. gondii-*infected 5xFAD mice.

### Myeloid cell activation and phagolysosomal-associated amyloid are increased in *T. gondii-*infected 5xFAD mice

**Fig. 2:**
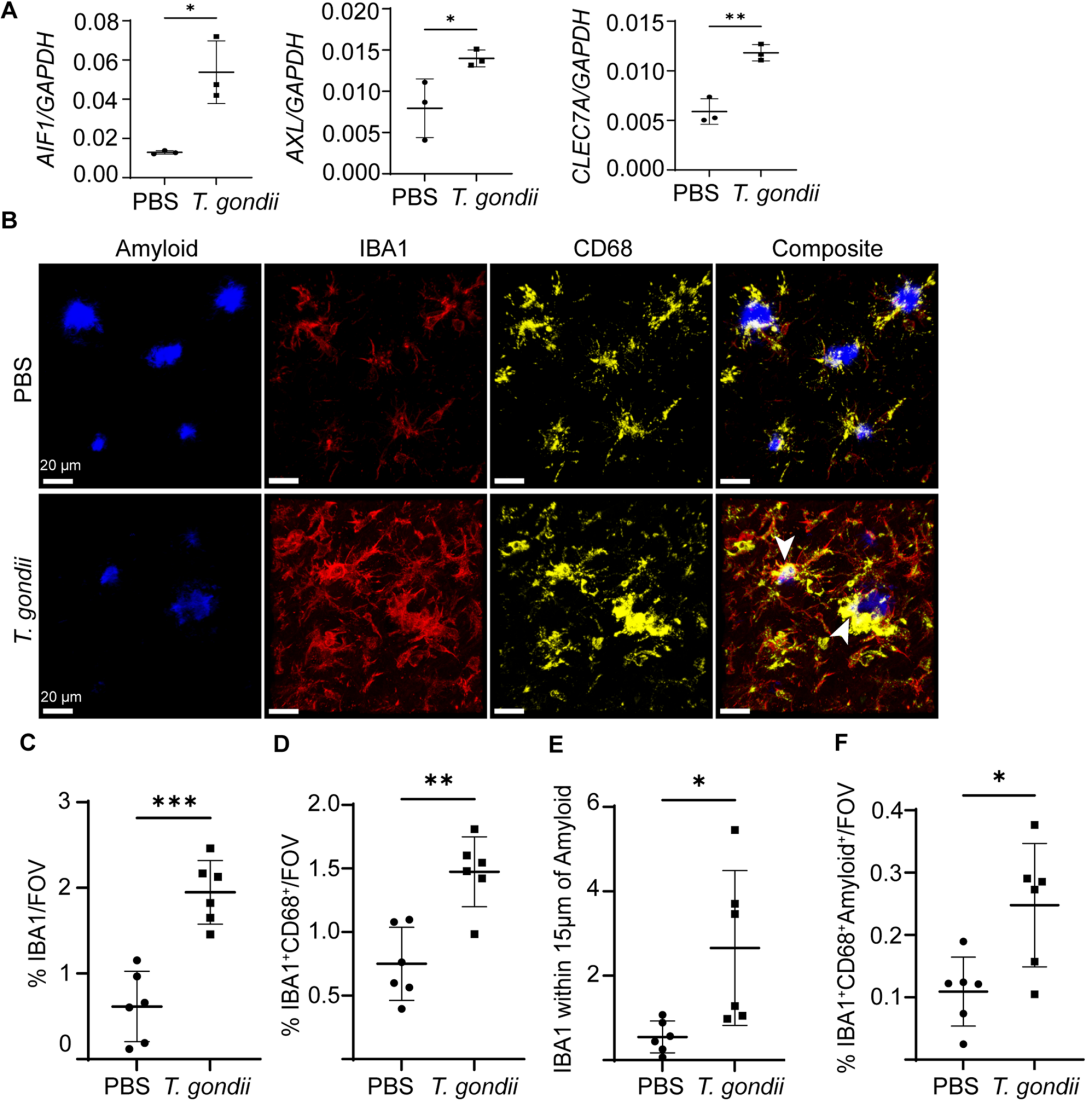
Colocalization of amyloid with myeloid and phagolysosomal markers increase in the cortex of *T. gondii*-infected mice. Brains were harvested from PBS-injected or *T. gondii*-infected 5xFAD mice and analyzed at 6 wpi. **A** qPCR for transcript levels of disease-associated microglia (DAM) markers AIF1, AXL, and CLEC7A normalized to GAPDH from one hemisphere of mouse brain tissue. **B** Representative images showing amyloid (Amylo-Glo), IBA1, CD68, and the merged channels (composite) in the cortex. Arrowheads indicate CD68 colocalization with amyloid cores. **C** Average percent of IBA1 volume per FOV in the cortex per mouse. **D** Average percent of CD68^+^IBA1^+^ volume per FOV of the cortex in each mouse. **E** Average IBA1 volume within a 15 µm radius of plaque cores was normalized to total plaque core volume per cortical FOV in each animal. **F **CD68 colocalization with amyloid in IBA1^+^ cells per cortical FOV in each animal. In A *n*=3 mice per group, in C-F *n*=6 mice per group. Student’s t test, *p<0.05, **p<0.01, ***p<0.001

Myeloid cells have been proposed to play an important role in regulating amyloid plaque size [7, 9] and are activated in the brains of *T. gondii-*infected AD mice [14, 15]. To determine the phenotype of brain-resident microglia and infiltrating myeloid cells during *T. gondii* infection of 5xFAD mice, we measured the levels of phagocytic disease-associated microglia (DAM) transcripts *AIF1* (IBA1), *AXL*, and *CLEC7a* [7]. These transcripts were elevated in the brains of *T. gondii*-infected 5xFAD mice at 6 wpi (Fig. 2A), suggesting an increase in gene expression associated with DAM. Based on these data, we sought to determine whether myeloid cells and phagocytic activity were increased in the brain during infection. We i.p. infected 5xFAD mice with *T. gondii* or injected PBS as a control and harvested brains for analysis at 6 wpi, as above. Fixed tissue sections were stained with antibodies to detect IBA1, a marker of myeloid cells, and CD68, a marker of phagosomes and activated microglia. Sections were also stained for amyloid with Amylo-Glo (Fig. 2B). We detected large IBA1^+^ cells around plaques in both PBS- and *T. gondii-*injected mice, as well as extensive CD68^+^ phagolysosomes that seemed to permeate to the center of amyloid plaques, specifically in the infected brains (Fig. 2B, arrowheads). FOVs in the cortex of each brain section were analyzed for the area of IBA1 and CD68 signal, which were significantly increased in the infected mice (Fig. 2C, Sup. Figure 1E). To determine the amount of CD68 within IBA1^+^ cells, we also quantified the colocalization of CD68 and IBA1 and found that CD68 signal within IBA1^+^ cells was increased (Fig. 2D), suggesting more phagolysosomes within IBA1^+^ cells in the cortex. These IBA1^+^ cells were also more likely to be within 15 μm of amyloid plaque cores following infection, suggesting close association of these cells to the plaque cores (Fig. 2E). We also detected an increase in amyloid within CD68^+^ phagolysosomes inside IBA1^+^ cells in the cortex (Fig. 2F, Sup. Figure 1 F). In the subiculum, myeloid cell area and localization near plaques were significantly increased in infected mice, but these cells did not have increased CD68^+^ phagolysosomes, nor was there increased amyloid within CD68^+^ phagolysosomes (Sup. Figure 1G-L). Altogether, these data suggest that chronic *T. gondii* infection increases phagolysosomal IBA1^+^ cells near plaques specifically in the cortex.

### MAC2^+^ cells are recruited to plaques during chronic infection

**Fig. 3:**
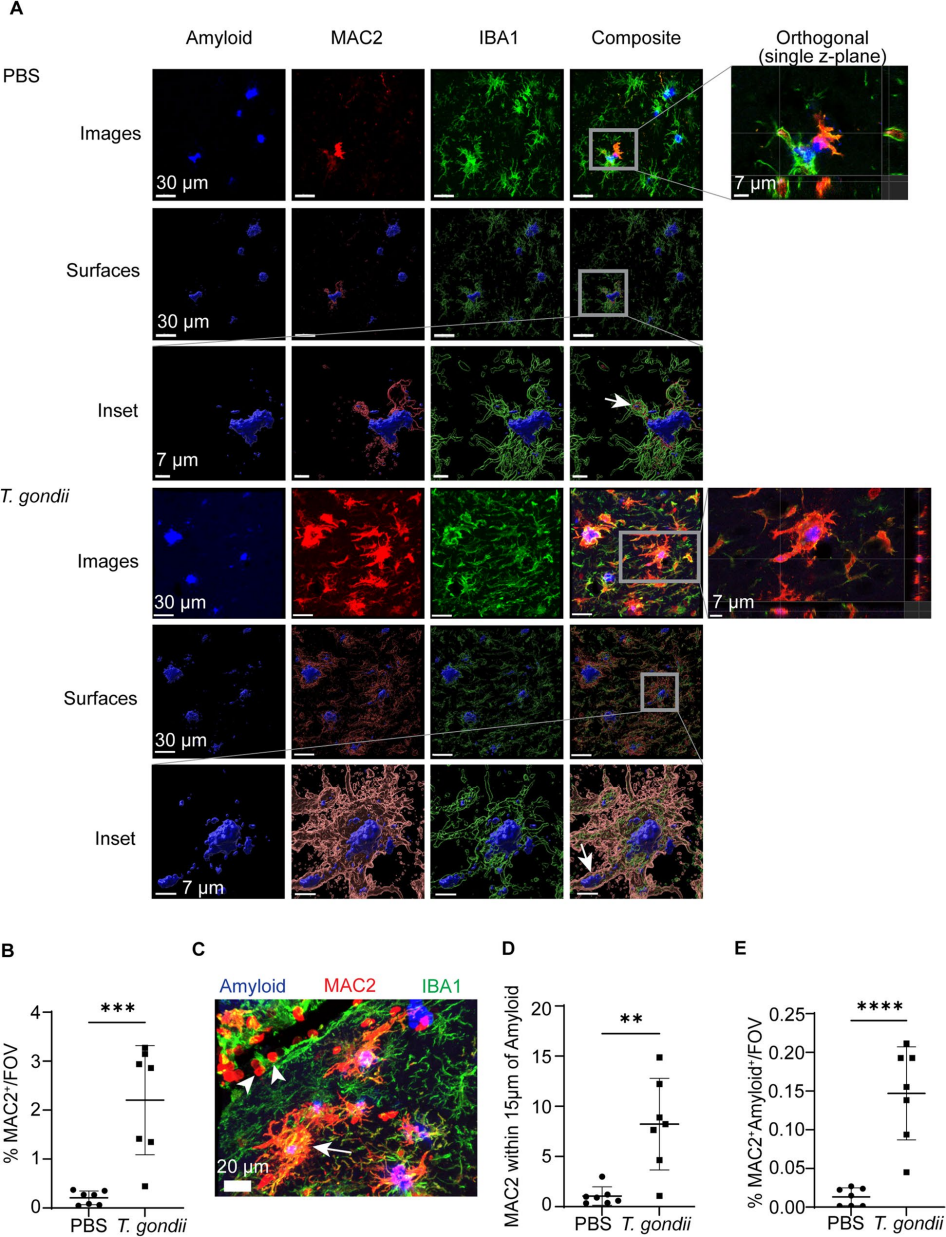
MAC2+ cells increase with infection and contain intracellular amyloid beta. 5xFAD mice were injected with PBS or infected with *T. gondii* for 6 weeks, and brain sections were imaged in the cortex using confocal microscopy. **A** Representative images of amyloid (Amylo-Glo), MAC2, and IBA1 staining and surfaces (rendered in Imaris). Insets with arrows depict amyloid within MAC2^+^IBA1^+^ cells. Orthogonal views with crosshairs on amyloid signal within the cells. **B** Average percent of MAC2 volume per FOV in the cortex per animal. **C** Example of MAC2^+^ cells (red) within a blood vessel (arrow heads) and surrounding amyloid plaques (blue, arrow). **D** Average MAC2^+^ volume within 15 µm of plaque cores, normalized to total plaque core volume per FOV per animal. **E** Average percent of MAC2 and amyloid core colocalization per FOV in the cortex per animal. In B, D, and E *n*=7 mice per group. Student’s t test, **p<0.01, ***p<0.001, ****p<0.0001

IBA1 is expressed on both brain-resident microglia and monocytes that infiltrate the brain from the bloodstream. To further characterize the IBA1^+^ cells surrounding plaques in the 5xFAD brain during chronic *T. gondii* infection, we examined MAC2 (Galectin-3), which is a marker of infiltrating monocytes [34], but can also be expressed on activated microglia [35–38]. Brain sections from control or *T. gondii-*infected 5xFAD mice at 6 wpi were stained for MAC2, IBA1, and amyloid. Most FOVs in PBS mice had little to no MAC2 signal; however, in PBS mice with detectable MAC2 signal, there was also more intense IBA1 signal (Fig. 3A, B). Compared to PBS-injected mice, there was a significant increase in MAC2^+^ cells in the brains of infected mice (Fig. 3A, Sup. Figure 2 A), and specifically in the cortex at 6 wpi (Fig. 3B). These data are consistent with our recent observation that MAC2^+^ cells are more abundant in the brains of C57BL/6 mice during *T. gondii* infection [39].

Since MAC2 may play a role in the ability of immune cells to phagocytose amyloid beta and regulate plaque burden [38], we sought to quantify the relationship between MAC2^+^ cells and amyloid cores. MAC2^+^ cells were found in the lumen of cortical blood vessels (white arrowheads), and in the parenchyma adjacent to amyloid plaques in the brain (white arrow) (Fig. 3C). We quantified MAC2^+^ cells within 15 μm of amyloid plaque cores and observed an increase in these cells near the plaques in the brains of infected mice (Fig. 3D). A 3D rendering of the z-stack and the orthogonal views of amyloid and MAC2 signal from infected and uninfected mice revealed that MAC2^+^ cells contained small specks of amyloid in both control and infected mice (Fig. 3A insets). The colocalization of amyloid with MAC2 was significantly higher following infection in both the cortex (Fig. 3E) and subiculum (Sup. Figure 2B), with some IBA1^+^MAC2^+^ cells in the cortex containing amyloid within CD68^+^ phagolysosomes (Sup. Figure 2 C), further indicating that infection increases myeloid cell recruitment to the brain and myeloid cell activation and interaction with amyloid plaques.

### Myeloid cells enter the infected brain during acute infection and remain elevated during chronic infection

**Fig. 4:**
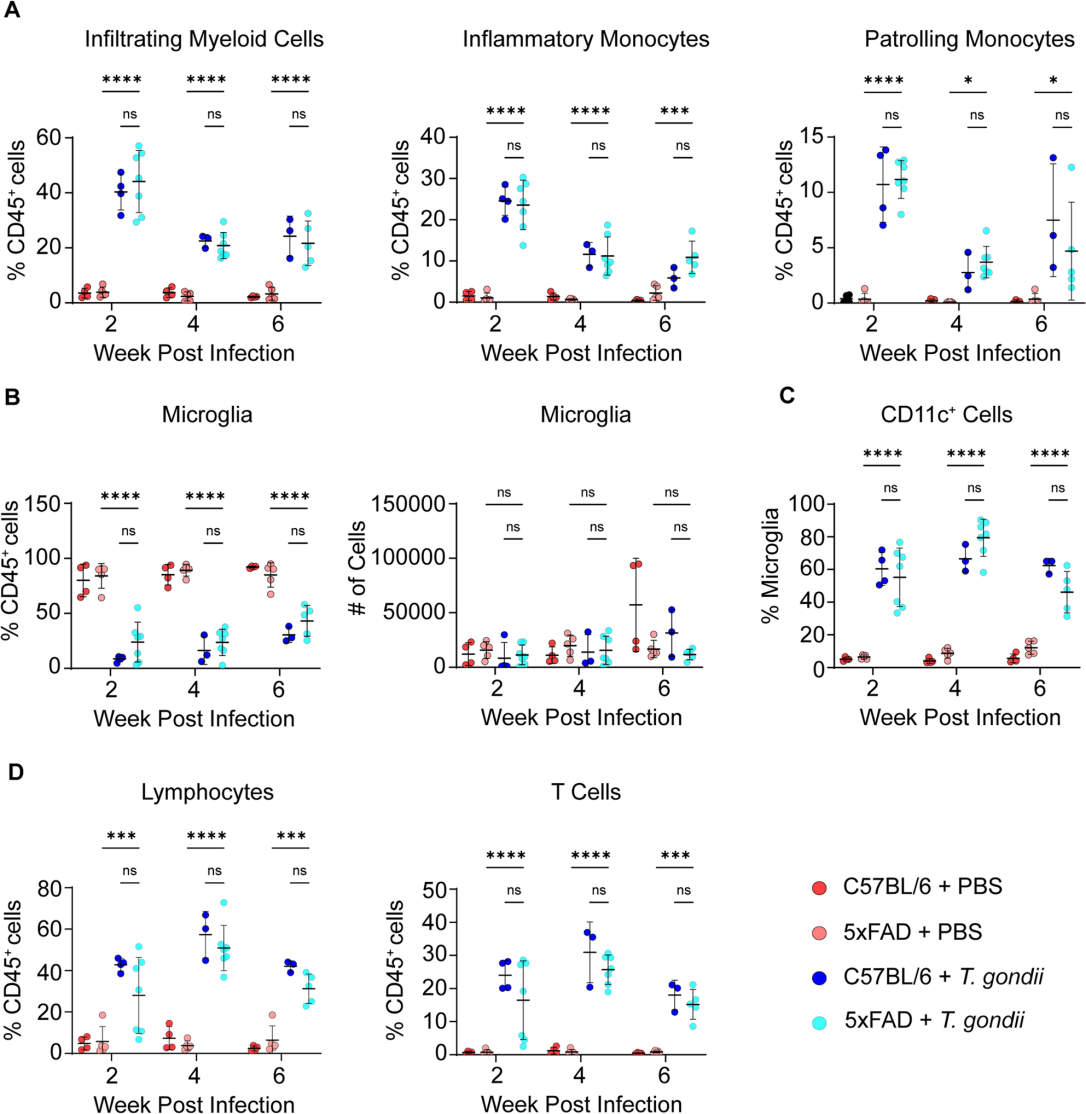
Infiltrating immune cells are recruited to the 5xFAD brain during acute *T. gondii* infection and persist through chronic infection. 5xFAD and C57BL/6 mice were injected with PBS or infected with *T. gondii*, and brain homogenates were analyzed by flow cytometry 2, 4, and 6 weeks later. **A** Infiltrating myeloid cells (CD45^hi^CD11b^+^), including patrolling (Ly6C^lo^) and inflammatory (Ly6C^hi^) monocytes as a percent of CD45^+^ cells. **B** Microglia (CD45^int^CD11b^+^) frequencies and absolute numbers. **C** CD11c^+^ cells (as a percent of all CD45^int^CD11b^+^ microglia). **D** Lymphocyte (CD45^+^CD11b^-^), and specifically T cell (CD3^+^) frequencies. In **A**-**D**, each dot represents one animal. *n*=3–8 mice per group. Two-way ANOVA followed by a post-hoc Tukey test, *p<0.05, ***p<0.001, ****p<0.0001, ns: not significant

To assess the kinetics of immune cell recruitment to the brain during *T. gondii* infection in 5xFAD mice, we compared the frequencies of infiltrating monocytes and lymphocytes and of brain-resident microglia at 2, 4, and 6 wpi in C57BL/6J and 5xFAD mice (Sup. Figure 3). Consistent with prior reports in C57BL/6J mice [39–42], the recruitment of infiltrating myeloid cells (CD45^hi^CD11b^+^), including both inflammatory (Ly6C^hi^CD45^hi^CD11b^+^) and patrolling monocytes (Ly6C^lo^CD45^hi^CD11b^+^), to the brain was highest at 2 wpi, and remained elevated even at 6 wpi in both genotypes of mice (Fig. 4A) [40]. Frequencies of microglia (CD45^int^CD11b^+^), as a percent of CD45^+^ cells, decreased as a result of the influx of immune cells during infection, but the total numbers of microglia remained the same in all conditions (Fig. 4B). CD11c^+^ microglia have been characterized as phagocytic disease-associated microglia (DAM) in the context of Alzheimer’s disease [7, 42]. Interestingly, CD11c^+^ cells within the CD45^int^CD11b^+^ “microglia” gate (Sup. Figure 3), also increased in the brains of infected C57BL/6J and 5xFAD mice, and remained highly elevated at 6 wpi (Fig. 4C). Frequencies of lymphocytes, and specifically T cells, were also increased at 2, 4, and 6 wpi, peaking at 4 wpi (Fig. 4D). Plasma levels of neurofilament light (NfL), a marker of axonal injury [43], were elevated at 2 and 4 wpi, coinciding with the influx of immune cells to the brain, before decreasing at 6 wpi (Sup. Figure 4A). Altogether, these kinetic changes suggest that immune cells were recruited to the brain in acute infection and may be associated with neuronal damage; however, as infection progressed to a chronic state, both the frequencies of persisting immune cells and the levels of circulating NfL declined.

### T cells increase in the cortex and are associated with myeloid cells and amyloid plaques during chronic infection

**Fig. 5:**
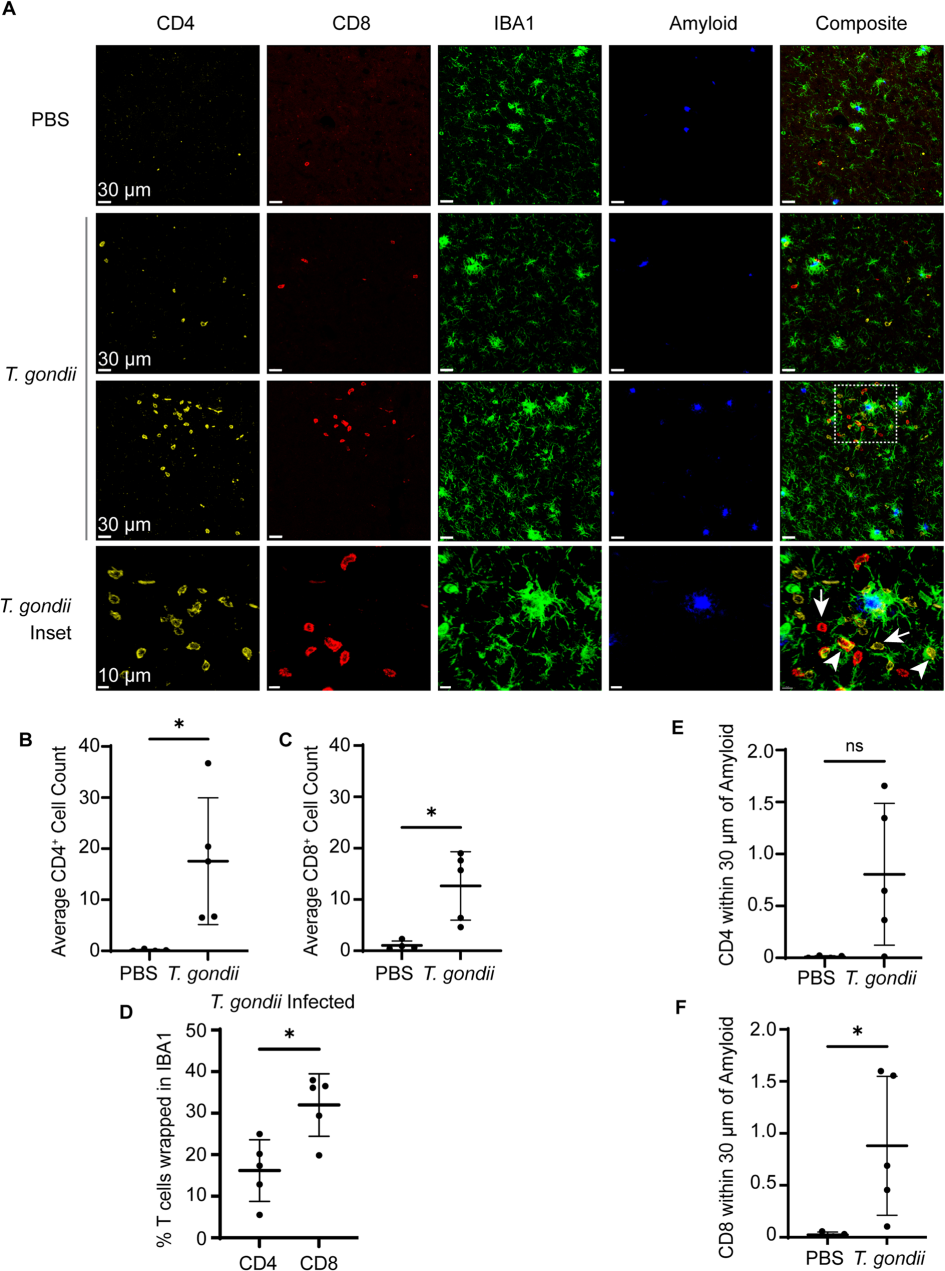
CD4^+^ and CD8^+^ T cells are increased in the brain during chronic infection and are associated with myeloid cells and amyloid. 5xFAD mice were injected with PBS or infected with *T. gondii* for 6 weeks, and brain sections were imaged in the cortex using confocal microscopy. **A** Representative images of CD4, CD8, IBA1, and amyloid staining in PBS and *T. gondii-* infected animals (top 2 rows). Third row shows CD4^+^ and CD8^+^ cells far from or near to IBA1^+^ cells during infection. Inset with arrowheads depict CD4^+^ and CD8^+^ cells closely associated with IBA1^+^ cells. Arrows depict CD4^+^ and CD8^+^ cells not associated with IBA1^+^ cells. **B** Average CD4^+^ cell count per FOV in the cortex per animal. **C** Average CD8^+^ cell count per FOV in the cortex per animal. **D** Average percent of T cells wrapped in IBA1 per FOV per animal in infected mice. **E** Average CD4^+^ volume within 30 µm of plaque cores, normalized to total plaque core volume per FOV per animal. **F** Average CD8^+^ volume within 30 µm of plaque cores, normalized to total plaque core volume per FOV per animal. In B-F *n*=4–5 mice per group. Welch’s t test, *p<0.05, ns: not significant

Although we found that CD3^+^ T cells increased in the 5xFAD mouse brain during chronic infection by flow cytometry, where these cells were located within the cortex, and whether they were associated with other cells or amyloid remained open questions. To address these points, we labeled CD4^+^ and CD8^+^ T cells, as well as IBA1^+^ myeloid cells and amyloid in the cortex of PBS or *T. gondii*-infected 5xFAD mice at 6 wpi (Fig. 5A). CD4^+^ and CD8^+^ T cells were found only rarely in the cortex of 5xFAD PBS-injected mice. In contrast, CD4^+^ and CD8^+^ T cells were significantly increased in the cortex during infection (Fig. 5A-C). In infected mice, CD4^+^ and CD8^+^ T cells were both closely associated with IBA1^+^ cells (Fig. 5A arrowheads) and found at a distance from IBA1^+^ cells (Fig. 5A arrows). To measure this, we quantified the percent of CD4^+^ or CD8^+^ T cells that were enwrapped in IBA1^+^ cells (defined as a T cell with *≥* 50% of the cell covered with IBA1). Although CD4^+^ T cells outnumbered CD8^+^ T cells in the brain by a ratio of 2:1, approximately 32% of CD8^+^ T cells and 17% of CD4^+^ T cells were enwrapped in IBA1 in the brains of infected mice (Fig. 5D), suggesting that CD8^+^ T cells were more likely to interact with myeloid cells compared to CD4^+^ T cells, despite fewer of these cells in the brain. In quantifying T cells within 30 μm to amyloid plaques, we found that CD8^+^ cells were also significantly enriched near plaques in the brains of infected mice (Fig. 5E and F).

### Peripherally derived immune cells mobilized from the skull are recruited to the chronically infected 5xFAD brain

**Fig. 6:**
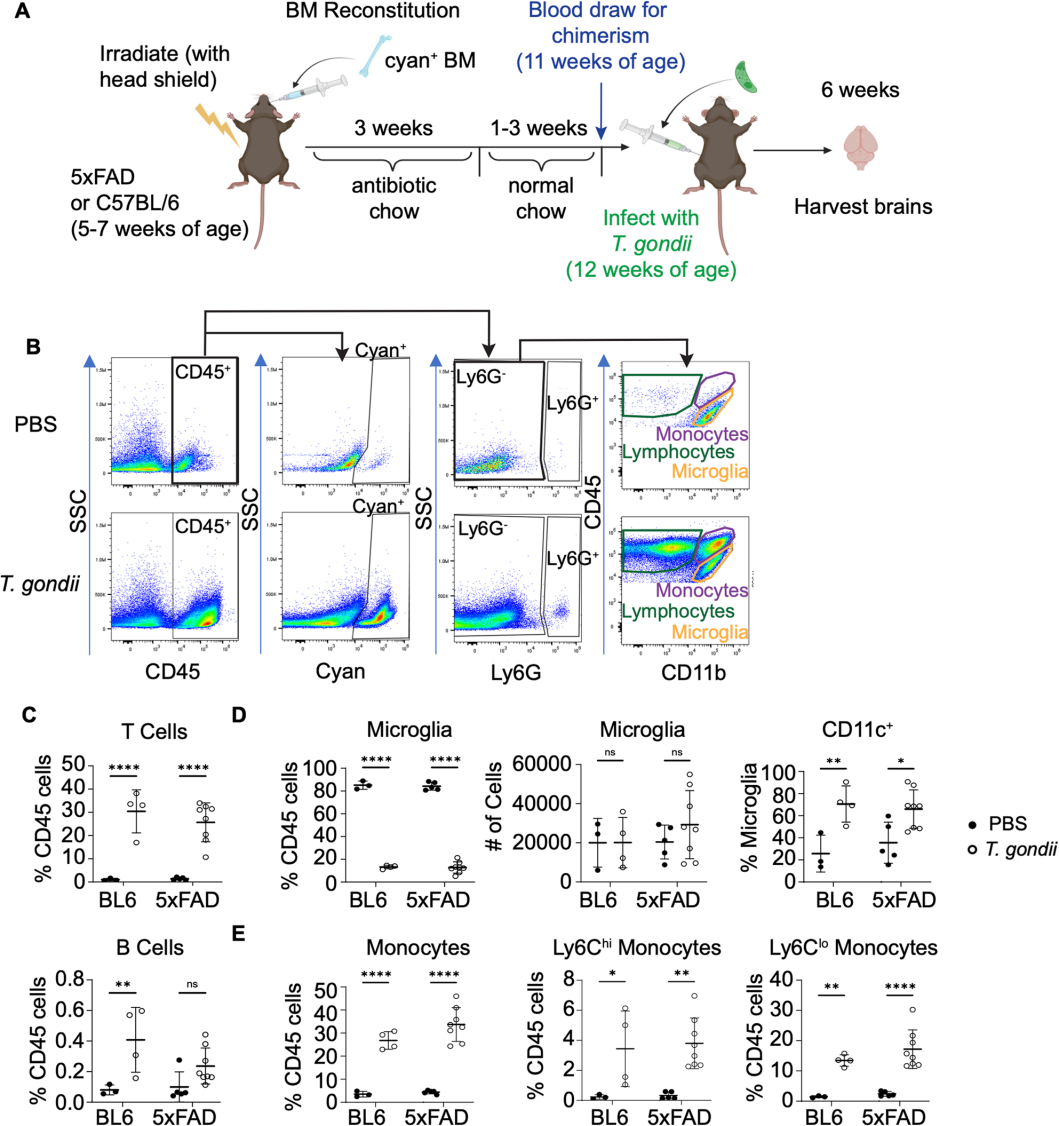
Infiltrating immune cells are recruited to the chronically infected brain. **A** Schematic depicting the bone marrow chimera and infection experiments. 5xFAD and C57BL/6 mice were irradiated with a head shield and reconstituted with CAG-CFP bone marrow cells before infection with *T. gondii*. Six weeks later, brains were collected and processed for flow cytometry. **B** Representative gating schema for cells from control and *T. gondii*-infected bone marrow chimeric mice. **C** T (CD3^+^) and B (CD19^+^) cell frequencies. **D** Microglia (CD45^int^CD11b^+^) frequencies and absolute numbers. CD11c^+^ cells as a percent of microglia. **E** Monocytes (CD45^hi^CD11b^+^), including Ly6C^hi^ and Ly6C^lo^ cells as a percent of CD45^+^ cells. In **C**-**E**, each dot represents a mouse. *n*=3–8 mice per group. Two-way ANOVA followed by Sidak’s test, *p<0.05, **p<0.01, ****p<0.0001, ns: not significant. Schematic created in BioRender (2025)

Since we observed increased infiltrating monocytes and activated microglia in the brains of chronically-infected 5xFAD mice, we sought to distinguish these cell types and their relationship with amyloid by conducting bone marrow chimera experiments. 5–7 week-old 5xFAD or C57BL/6J mice were irradiated with a headshield to reduce the likelihood of disruption of the blood-brain barrier [44, 45] and reconstituted with bone marrow cells from mice expressing cyan fluorescent protein (CFP) ubiquitously (CAG-CFP mice) (Fig. 6A). Chimerism in peripheral blood was examined by flow cytometry for cyan^+^ granulocytes at 11 weeks of age and averaged 85.17% in the C57BL/6 mice and 86.54% in the 5xFAD mice (Sup. Figure 5 A), indicating that there was no difference in the chimerism rate between the genotypes. The C57BL/6 and 5xFAD bone marrow chimera mice were i.p. injected with PBS or infected with *T. gondii* as above, at 12 weeks of age, and their brains were analyzed at 6 wpi (Fig. 6A). We first confirmed that the bone marrow transplant did not affect the frequencies of the immune cell populations previously observed in the brain at 6 wpi. Flow cytometry was used to identify infiltrating cells (cyan^+^), neutrophils (Ly6G^+^), T cells (CD3^+^), B cells (CD19^+^), NK cells (NK1.1^+^), NKT cells (CD3^+^NK1.1^+^), monocytes (LyC6^hi^ and LyC6^lo^), and microglia (CD45^int^CD11b^+^) (Fig. 6B, Sup. Figure 4B). The frequencies of T cells, CD11c^+^ cells, and Ly6C^hi^ and Ly6C^lo^ monocytes were increased in C57BL/6 and 5xFAD chimeric mice following infection (Fig. 6C-E), similar to the infected nonchimeric C57BL/6 and 5xFAD mice (Fig. 4). There was also no significant effect of genotype on any of the cell populations identified by flow cytometry. Infected chimeric mice lost weight around 10–12 days post-infection and gradually improved with time (Sup. Figure 4B). These data recapitulate previously published weight loss in infected C57BL/6 mice [40]. At the 41 dpi endpoint, there was a significant difference between uninfected and *T. gondii* infected chimeric 5xFAD mice (Sup. Figure 4B).

**Fig. 7:**
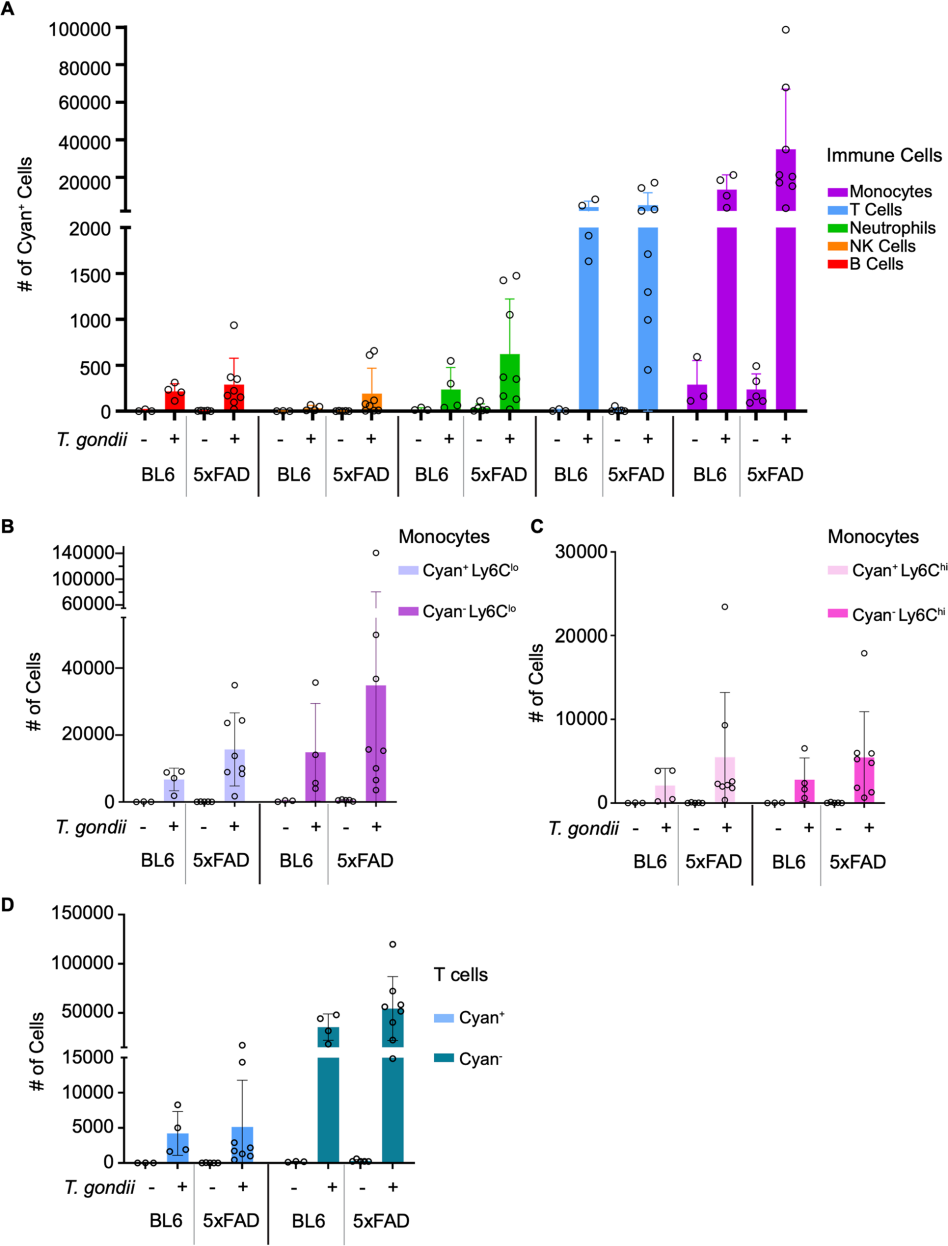
Brain-infiltrating immune cells are predominantly derived from the skull bone marrow during *T. gondii* infection. Flow cytometry analysis of brain homogenates from C57BL/6 and 5xFAD mice transplanted with CFP+ bone marrow and injected with PBS or infected with *T. gondii* for 6 weeks. These cells were identified based on the gating strategy shown in Figure 6B. **A** Number of cyan^+^ cells by each immune cell population in control or infected C57BL/6 or 5xFAD mice. **B**,** C** Number of cyan^+^ and cyan^-^ Ly6C^lo^ (B) or Ly6C^hi^ (C) monocytes (CD45^hi^CD11b^+^) in control or infected C57BL/6 or 5xFAD mice. **D** Number of cyan^+^ and cyan^-^ T cells (CD3^+^) in control or infected C57BL/6 or 5xFAD mice. *n*_B6_= 3–4 mice per group, *n*_5xFAD_=5–8 mice per group

Channels connecting the skull bone marrow to the dura have recently been described and allow myelomonocytic cells to gain direct access to the brain parenchyma by bypassing the blood [46–48]. The discovery of these channels has prompted interest in the role of skull bone marrow-derived cells in homeostasis, neurodegeneration, and infection [48]. For these bone marrow transplantation experiments, we head-shielded the C57BL/6 and 5xFAD mice during irradiation, such that the chimeric mice maintained endogenous skull bone marrow, whereas the peripheral bones were reconstituted with cyan^+^ bone marrow. As a result, this system can be used to identify cells from peripheral bone marrow origins below the neck. We used flow cytometry to quantify the numbers of infiltrating immune cells in the brain following infection and to determine the extent to which these cell populations were cyan^+^. *T. gondii* infection induced a robust infiltration of cyan^+^ immune cells into the brains of both 5xFAD and C57BL/6 mice (Fig. 7A). Among the cyan^+^ immune cells, most were monocytes, followed by T cells (Fig. 7A). In examining the monocyte populations in the infected 5xFAD brains, 31% of the Ly6C^lo^ monocytes and 50.1% of the Ly6C^hi^ monocytes were cyan^+^ (Fig. 7B-C). On average, a higher number of Ly6C^lo^ monocytes in the chronically infected 5xFAD mouse brain were cyan^−^, suggesting that they were derived from the skull bone marrow niche. Among T cells, only 8.6% of the total T cell population in the infected 5xFAD brain were cyan^+^ (Fig. 7D), indicating that most of the T cells were from the head-shielded skull bone marrow and not from peripheral bone marrow niches. While the majority of the infiltrating immune cells were cyan^−^, suggesting skull bone marrow origin, we did find a substantial number of infiltrating immune cells that were cyan^+^, suggesting a more peripheral origin.

In order to determine whether peripherally derived and skull derived cells differed in their expression levels of standard phenotyping markers, we compared the mean fluorescence intensity (MFI) of key markers between cyan^+^ and cyan^−^ monocytes and T cells in PBS-injected and *T. gondii*-infected mice (Sup. Figure 6). The only population of infiltrating immune cells that significantly differed in expression were cyan^+^ and cyan^−^ T cells in *T. gondii-*infected 5xFAD brains: the MFI of CD3 on cyan^+^ T cells was higher than on cyan^−^ T cells (Sup. Figure 6A). These data suggest that, at least in our limited flow panel, skull-derived and more peripherally-derived infiltrating immune cells did not appear to differ substantively in their expression of standard phenotyping markers.

### Peripherally-derived myeloid cells are recruited to amyloid plaques during chronic infection

**Fig. 8:**
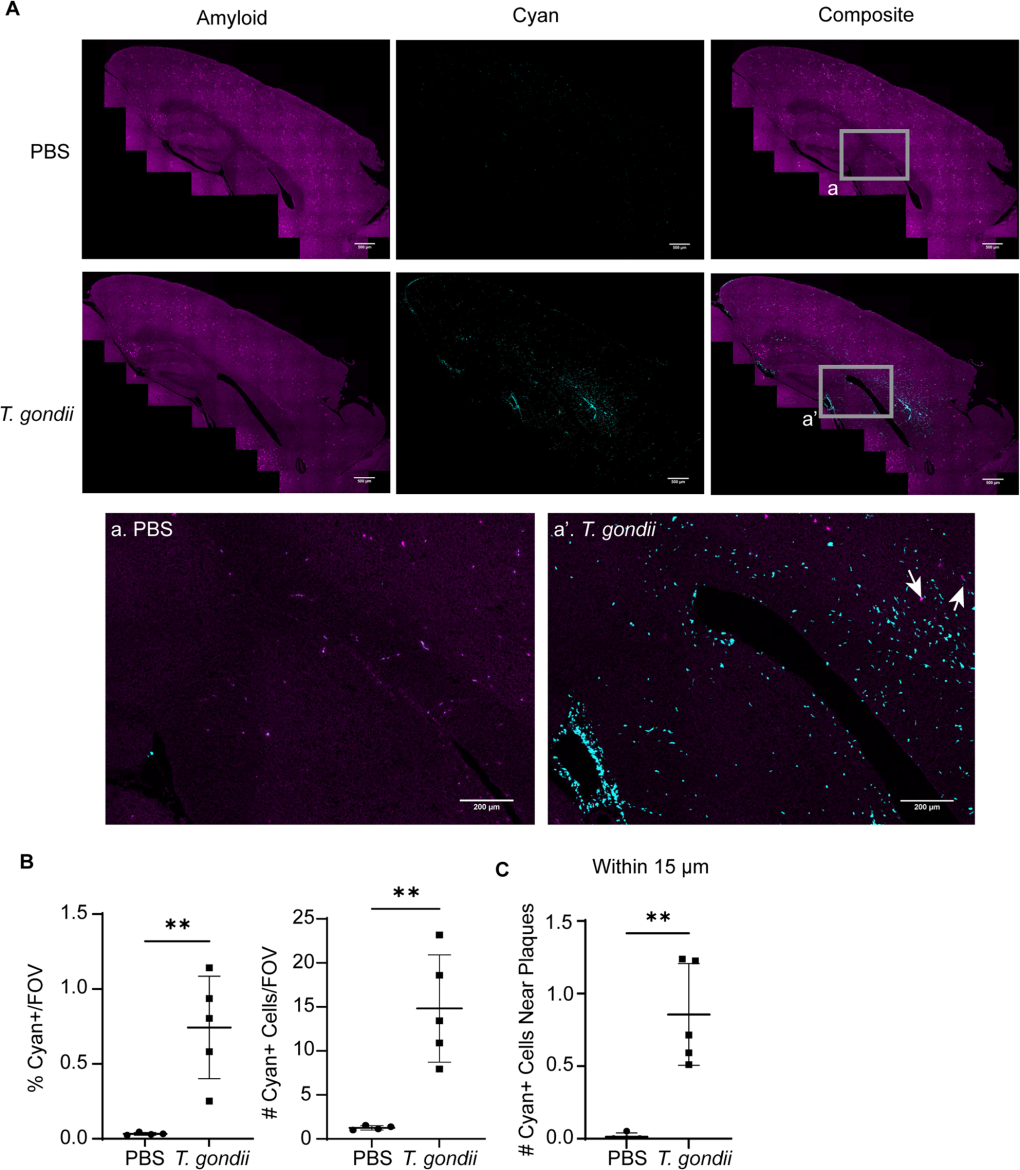
Peripherally-derived immune cells are recruited to plaques in the brains of chronically infected 5xFAD mice. Chimeric 5xFAD mice were injected with T. gondii or PBS as a control for 6 weeks, and the brain sections were imaged using confocal microscopy. **A** Representative images depict amyloid (Amylo-Glo, magenta), and cyan+ cells in PBS and T. gondii-infected brains. a. Magnified inset from the PBS-injected mouse. a'. Magnified inset from the T. gondii-injected mouse. Arrows indicate cyan+ cells near plaques. **B** Average percent area of cyan+ cells/FOV in each mouse. **C** Average number of cyan cells/FOV in the cortex per mouse. **D** Average numbers of cyan+ cells within 15 µm of plaques, normalized to the number of plaques, and displayed per animal. *n*=4-5 mice per group. Student’s t test, **p<0.01

In order to determine where the cyan cells localized in the brain tissue, we stained brain sections for amyloid (Amylo-Glo) and visualized cyan^+^ cells and amyloid plaques in tilescans of the cortex (Fig. 8A). The irradiation and bone marrow transplant procedure did not affect the levels of amyloid in the chimeric 5xFAD mice (Sup. Figure 7). In PBS-injected 5xFAD chimeric mice, there were very few peripherally-derived cells in the brain parenchyma (Fig. 8A, top row). In contrast, in the brains of *T. gondii-*infected 5xFAD bone marrow chimeric mice at 6 wpi, peripherally-derived cyan^+^ immune cells were found throughout the cortex, hippocampus, and ventricles, with some cells colocalizing with plaques, and others localized near the ventricles (Fig. 8A, inset and arrows). Quantification of the cyan^+^ cells throughout the cortex of *T. gondii-*infected and control mice showed that infected mice had significantly greater cyan^+^ area and increased numbers of cyan^+^ cells in the brain parenchyma (Fig. 8B). The cyan^+^ cells within 15 μm of plaques were also increased after *T. gondii* infection (Fig. 8C).

**Fig. 9:**
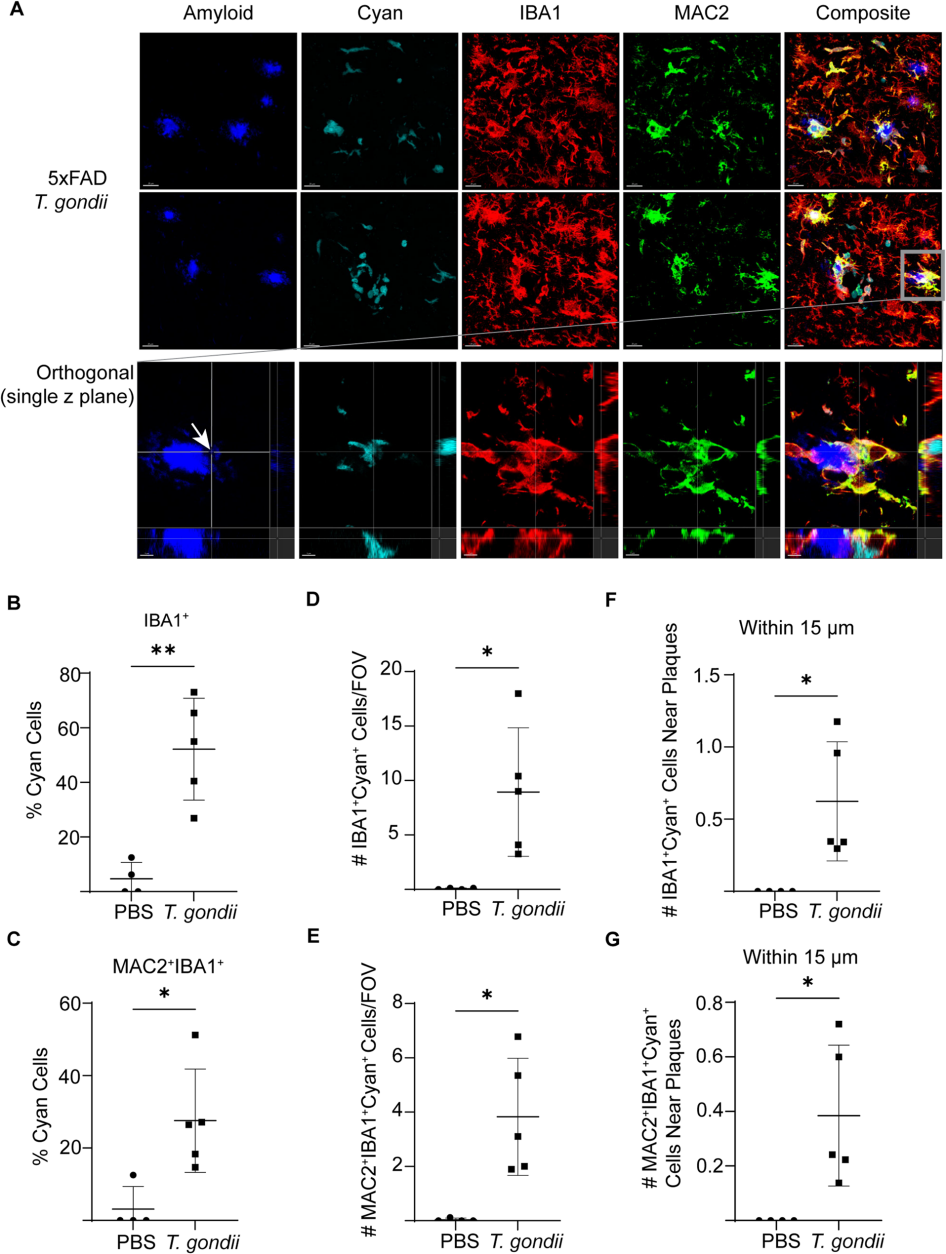
Peripherally-derived IBA1^+^MAC2^+^ myeloid cells are recruited to amyloid plaques in *T. gondii*-infected mice during chronic infection. Chimeric 5xFAD mice were injected with *T. gondii* or PBS as a control for 6 weeks, and the brain sections were imaged using confocal microscopy. **A** Representative images of amyloid plaques (Amylo-Glo), cyan^+^ cells, IBA1, and MAC2 in the brains of *T. gondii*-infected 5xFAD chimeric mice. Inset depicts magnified and orthogonal views of an IBA1^+^MAC2^+^cyan^+^ cell containing amyloid signal at the crosshairs (arrow). The average percent of cyan^+^ cells that were IBA1^+^ (**B**) and IBA1^+^MAC2^+^ (**C**) per FOV. **D** The average number of IBA1^+^cyan^+^ cells in each FOV and (**F**) within 15 µm of plaques (normalized to the number of plaques) per FOV in the cortex. **E** The average number of IBA1^+^MAC2^+^cyan^+^ cells in each FOV and (**G**) within 15 µm of plaques (normalized to the number of plaques) in each FOV in the cortex. *n*=4–5 mice per group. Student’s t test, *p<0.05, **p<0.01

To further characterize the peripherally-derived cyan^+^ cells, we stained brain sections from infected 5xFAD mice for amyloid (Amylo-Glo), IBA1, and MAC2 (Fig. 9A). In the brains of infected mice, we observed many clusters of cyan^+^ cells near plaques in the cortex (Fig. 9A) and some cyan^+^ cells with small volumes of amyloid inside their cell bodies (Fig. 9A inset, Sup. Movie 1). Approximately 50% of the cyan^+^ cells in infected mice also expressed IBA1, indicating that they were myeloid cells, and 30% of the cells were IBA1^+^MAC2^+^, suggesting that they were activated (Fig. 9B and C). We next quantified the differences in these double- and triple-positive cell populations near plaques. IBA1^+^cyan^+^ cells and MAC2^+^IBA1^+^cyan^+^ cells were significantly increased in the cortex after infection (Fig. 9D and E). The infiltrating myeloid cells within 15 μm of plaques were also increased after infection (Fig. 9F and G). Altogether, these data show that infection induced an increase in peripherally-derived myeloid cells near plaques, and some of these cells contained amyloid beta.

## Discussion

Like moths drawn to light, the recruitment of microglia to amyloid plaques in Alzheimer’s disease is an observation harkening back to Alois Alzheimer’s initial notes on AD pathology and remains a major pathological finding in the disease. It is now appreciated that microglia play important roles in plaque clearance and formation. However, many open questions remain, including how peripheral immune cells and infection may contribute to or antagonize this process. Herein, we build on the work of several labs in the field and investigate the observation that chronic infection with *T. gondii* decreases amyloid plaques in the brains of AD model mice [13–15, 28]. Indeed, we observed reduced amyloid core volume and less spherical plaque formation in *T. gondii-*infected 5xFAD mice. We also detected increased CD8^+^ T cells and elevated myeloid cells with amyloid within their phagolysosomes near plaques during infection, and determined that some of these myeloid cells are peripherally derived and express MAC2.

Historically, brain-resident microglia and monocytes recruited to the brain parenchyma have been difficult to distinguish from each other. Evidence suggests that monocytes can engraft into the same niche as microglia when microglia are removed by CSF1R targeted deletion, CSF1 inhibition, or irradiation [44, 45]. In the context of neurodegeneration, some recruited myeloid cells express similar transcripts as microglia (and were described as DAMs but later identified as disease inflammatory macrophages/DIMs), although there also remains a subset of monocytes that take on a distinct profile [8]. Functionally, both monocytes and microglia appear to be capable of phagocytosing amyloid beta and of antigen presentation [7, 44, 49, 50]. In AD mouse models, most myeloid cells in the brain parenchyma and near plaques are microglia [51, 52], though some studies have shown that a small percentage of the myeloid cells near plaques in AD may be peripherally derived [8, 53]. In our data, there were few to no peripherally-derived cells near plaques of uninfected 5xFAD mice. Yet, during infection, these cells increased dramatically near plaques. Additionally, when myeloid cells were recruited to plaques, we observed an increase in amyloid within phagolysosomes and an overall reduction in amyloid burden. By flow cytometry, we were also able to determine that many microglia expressed CD11c^+^, a classic DAM marker that was also recently described in a reparative, beneficial microglia subtype [43]. It is possible that interactions between infiltrating monocytes and brain-resident microglia in the AD brain may enhance the activities of microglia or influence their function.

Finally, an additional question is how other immune cells interact with myeloid cells to influence amyloid beta pathology. T cells, NK cells, and B cells have been suggested to play a role in enhancing microglia clearance of amyloid beta [54]. Additionally, pre-immune IgG can alleviate amyloid burden in RAG-deficient mice [54], suggesting that the B cell response against amyloid beta may be beneficial in amyloid removal. More recently, CD8^+^ T cells were found to directly interact with microglia via CXCR6-CXCL16 binding to promote microglia clearance of amyloid plaques [55]. Interestingly, neutrophils may have a more detrimental effect, since the presence of these cells in the brain is associated with elevated amyloid [56]. Thus, although myeloid cells may control amyloid dynamics, it is increasingly apparent that other immune cells contribute to the neuroimmune response to amyloid beta in Alzheimer’s disease.

Non-myeloid peripherally-derived immune cells were also observed near plaques in the brains of infected mice in our studies. In particular, *T. gondii* induces a robust T cell infiltration of the brain parenchyma, and T cells can interact with microglia and other immune cells to influence amyloid and tau levels [57, 58]. It is possible that *T. gondii*-specific T cells recruited to the brain following infection may have cross-reactivity for amyloid via shared epitopes, similar to T cells in multiple sclerosis patients that may have cross reactivity to Epstein-Barr virus nuclear antigen 1 and myelin antigens [57]. Alternatively, T cells may directly interact with microglia and monocytes in the brain to enhance their function. Previous research has shown that T cells are present in the parenchyma of AD mice and increased after *T. gondii* infection with type II strain parasites, which cause a reduction in amyloid [13]. Indeed, we observed an increase in both CD4^+^ and CD8^+^ T cells interacting with IBA1^+^ cells in infected mice, and CD8^+^ T cells were also found near amyloid in infected 5xFAD mice. However, it is unlikely that T cells alone are responsible for the reduction in amyloid, since type III strain parasites also induce a strong T cell response but fail to reduce amyloid burden [13]. Future studies to determine the role of T cells in mediating myeloid cell activity and amyloid dynamics may be particularly useful.

In this study, we refer to “peripherally-derived cells” in the bone marrow transplantation experiments as those from bones below the skull. Since we used head shielding during lethal radiation prior to transplantation of cyan^+^ bone marrow, we were able to discern both cyan^−^ and cyan^+^ immune cells in the brains of mice after infection. In particular, we infer that the cyan^−^ monocytes and T cells may be from skull bone marrow. It has been proposed that myeloid cells trafficking through channels from the skull to the brain parenchyma may serve as an additional layer of protection for the CNS [46]. Although we did not find major differences between cyan^+^ and cyan^−^ cells in their expression of standard phenotyping markers by flow cytometry, it is possible that a more in-depth analysis of a broader array of protein markers or RNA transcripts would reveal differences between these cell populations. Our analysis may also include some peripherally-derived cells within the skull bone marrow, as it has been suggested that up to 40% of the skull bone marrow niche can be populated by donor-transplanted cells 4 weeks after reconstitution [46]. While it remains unclear what role skull-derived immune cells play in the overall response to amyloid during *T. gondii* infection, this may be an important future question with therapeutic benefit for the fields of both neurodegeneration and infectious diseases.

Though infection with *T. gondii* in Alzheimer’s model mice results in persistent decreases in amyloid plaque burden, it is unlikely that unmodified *T. gondii* infection would be of therapeutic benefit to prevent or treat Alzheimer’s disease, due to infection associated neuronal damage or the potential for re-activation of latent infection. *T. gondii* infection of neurons can result in increased neuronal death and affects neuronal polarization and extracellular vesicle secretion [59–61]. Acute infection also damages the blood brain barrier in mice, though this is restored during chronic infection [62]. We observed an increase in neuron axonal damage, as measured by plasma NfL levels, which temporally coincided with the height of immune cell recruitment to the brain at 2 and 4 wpi. By 6 wpi, plasma NfL declined in infected mice, though peripheral immune cells were maintained in the brain. This uncoupling of neuronal damage and infiltrating immune cells may signal a change in the function of the responding immune cells, as infection progresses from acute to chronic stage. It should also be noted that *T. gondii-*infected C57BL/6 mice maintain a higher degree of neuronal inflammation than humans during chronic infection, though longer temporal studies in mice appear to suggest that mice do not have significant long-term effects of *T. gondii* infection [63]. However, the impact of acute infection should not be overlooked. Additionally, reactivated *T. gondii* infection in immunocompromised humans and mice can result in severe neurological problems and even death [64, 65].

Although there are significant concerns associated with developing therapeutics based on *T. gondii* infection, our results suggest that persistent immune cell infiltration of the brain is associated with reduced amyloid. Whether this effect is due to preventing new plaque formation or decreasing the size of existing plaques remains unclear. Current therapies are being developed that target amyloid via macrophage and T cell chimeric antigen receptor cells, with promising results [66, 67]. Other potential therapeutic options include iPSC-derived microglial delivery of amyloid degradation enzymes like neprilysin [68]. A combinatorial approach that limits concerns and uses both the parasite and the neuroimmune response to reduce amyloid could include an attenuated, or “kill-switch” enabled *T. gondii* strain that directly (through injection of therapeutic proteins) primes myeloid or other immune cells to target amyloid, as this has been demonstrated to be possible in neurons [69]; however, further studies would be needed to optimize this approach.

## Conclusions

Altogether, our data support previous reports that myeloid cells, and in particular, peripherally-derived monocytes enter the brain and may contribute to reducing amyloid pathology in Alzheimer’s disease mice. Although it is unlikely that infection with *T. gondii* is beneficial for overall brain health, understanding the origin of the responding immune cells and how these cells can impact pathology in the brain provides an important framework for the development of future therapeutics, such as chimeric antigen receptor cells targeting amyloid [66, 67], which rely upon immune cell mobilization to the brain to prevent or treat AD. Future studies on AD pathology and infection in humans may help us understand how anti-amyloid immune responses are generated and may eventually enhance AD drug development.

## Supplementary Information

Supplementary Material 1: Movie S1. Amyloid signal is detectable inside IBA1^+^cyan^+^ cells. This movie represents the inset image from Fig. 9A. Chimeric 5xFAD mice were injected with T. gondii, and 6 weeks later the brain sections were imaged using confocal microscopy for amyloid, IBA1, and cyan^+^ cells. Surfaces were generated for each channel in Imaris, and amyloid signalis within a cell that is both IBA1^+^and peripherally derived.

Supplementary Material 2: Sup. Fig 1: Amyloid and IBA1^+^ cells in the subiculum *T. gondii*-infected 5xFAD animals. Three-month-old 5xFAD mice were injected i.p. with PBS or type II *T. gondii*, and amyloid and IBA1^+^ cells were analyzed at 6 wpi. A) Representative images show amyloid (Amylo-Glo) signal in the subiculum of the hippocampus of PBS or *T. gondii*-injected mice. B) Average percent of amyloid area per FOV in each mouse. C) Average volume of amyloid plaque cores per FOV. D) Average numbers of amyloid plaques per FOV. E-F) Average percent of CD68 signal per FOV (E) and (F) CD68^+^amyloid^+^ signal per FOV in the. G-I) Average IBA1 (G), CD68 (H), and IBA1^+^CD68^+^ signal per FOV. J) IBA1 volume within a 15 µm radius of plaque cores was normalized to total plaque core volume per FOV in each animal. K-L) CD68 colocalization with amyloid (K) and in IBA1^+^ cells (L) per FOV in each animal. For B-L, *n*= 6-7 mice per group. Student’s t test, * *p*<0.05, **p<0.01, ns: not significant. Sup. Fig. 2: MAC2^+^ cells increase in the subiculum and some MAC2^+^ cells in the cortex contain amyloid. 5xFAD mice were injected with PBS or infected with *T. gondii* for 6 weeks, and markers for myeloid cell activation and amyloid were imaged in the subiculum of the hippocampus or cortex. A) Average percent of MAC2 volume per FOV in the subiculum per animal. B) Average percent of MAC2 and amyloid core colocalization per FOV in the subiculum per animal. *n*=7 mice per group. Student’s t test, **p<0.01, ***p<0.001. C) Representative FOV of amyloid colocalizing with CD68 (arrow head) in an IBA1^+^MAC2^+^ cell as seen in 3D view (top row) and inset orthogonal views (bottom rows). Sup. Fig. 3: Gating schema for infiltrating immune cells at 2, 4 and 6 wpi. 5xFAD and C57BL/6 mice were injected with PBS or infected with *T. gondii*, and brain homogenates were analyzed by flow cytometry. Representative gating scheme for quantifying lymphocytes (CD11b^-^CD45^+^), monocytes (CD45^hi^CD11b^+^), and microglia (CD11b^+^CD45^int^). Lymphocytes were further defined as CD3^+^ T cells, monocytes as Ly6C^hi^ or Ly6C^lo^ monocytes, and microglia as CD11c^+^. Sup. Fig. 4: Mice infected with *T. gondii* display systemic changes in neurofilament light and weight loss. A) 5xFAD mice were injected with PBS or infected with *T. gondii*, and levels of neurofilament light (NfL) were measured within plasma collected at 2, 4 and 6 weeks post-infection. *n*=2-8 per group B) Chimeric 5xFAD and C57BL/6 mice were injected with PBS or infected with *T. gondii* and weighed from 0-41 days post infection. % weight change from day 0 was calculated for each mouse and averaged across mice from days 2-6, 7-9, 10-12, 13-15, 16-21, 22-28, 29-35, 36-41. Endpoints compared using Student’s t test. *n*=4-9 mice per group. Sup. Fig. 5: Infiltrating immune cells are recruited to the chronically infected brain. 5xFAD and C57BL/6 mice were irradiated with a head shield and reconstituted with CAG-CFP bone marrow cells before infection with *T. gondii*. Six weeks later, brains were collected and processed for flow cytometry. A) Percent of cyan^+^ neutrophils in the peripheral blood of C57BL/6 (BL6) or 5xFAD mice at 11 weeks after bone marrow transplant as a measure of blood chimerism. B) Representative gating schema for cells from control and *T. gondii*-infected bone marrow chimeric mice. Sup. Fig. 6: Mean fluorescence intensity (MFI) of phenotyping markers on cyan- and cyan^+^ T cells and monocytes in the brain. 5xFAD and C57BL/6 mice were irradiated with a head shield and reconstituted with CAG-CFP (cyan) bone marrow cells before infection with *T. gondii*. Six weeks later, brains were collected and processed for flow cytometry. A) CD3 expression as measured by geometric mean fluorescence intensity (MFI) for cyan^+^ and cyan^-^ CD3^+^ T cells. (B) CD45 and (C) CD11b MFI expression in CD11b^+^CD45hi monocytes. Ly6C MFI expression in (D) CD11b^+^CD45^hi^Ly6C^lo^ monocytes and (E) CD11b^+^CD45^hi^Ly6C^hi^ monocytes. CD11c MFI expression in (F) CD11b^+^CD45^hi^Ly6C^lo^ monocytes and (G) CD11b^+^CD45^hi^Ly6C^hi^ monocytes. In A-G, each dot represents one animal. *n*=3-8 mice per group. Two-way ANOVA followed by a post-hoc Fisher’s LSD, **p<0.01, ns: not significant. Sup. Fig. 7: Amyloid burden in uninfected 18-week-old 5xFAD mice with and without bone marrow transplantation. 5xFAD (unirradiated, untransplanted) and chimeric 5xFAD (irradiated and transplanted with CAG-CFP bone marrow) mice were injected with PBS at 12 weeks of age and analyzed 6 weeks later. A) Representative images from the cortex and subiculum of mice without (left) and with (right) irradiation and peripheral bone marrow reconstitution. B) The percent area of amyloid per FOV and the average plaque size in control and irradiated mice. In A and B, data reflect averages of 1-4 FOVs per section, 2 sections per animal. n_5xFAD_ normal = 6, n_5xFAD irradiated_ = 4. Student’s t test, ns: not significant.

## Data Availability

All data supporting the conclusions of this article are included within the paper and its Supporting information files.

## Abbreviations

AD: Alzheimer’s Disease
DAMs: Disease-Associated Microglia
IBA1: Ionized Calcium Binding Adapter Molecule-1
MAC2: Macrophage-2 Antigen
Thio: S-Thioflavin-S
