# Supplementary material for: Toxoplasma gondii drives myeloid immune cell recruitment to amyloid plaques in Alzheimer’s model mice
Source: J Neuroinflammation. 2026 Jan 17;23:64. doi: 10.1186/s12974-025-03666-2 (PMC12896096; doi:10.1186/s12974-025-03666-2)
Supplement: Supplementary file 2 — Supplementary Material 2: Sup. Fig 1: Amyloid and IBA1+ cells in the subiculum T. gondii-infected 5xFAD animals. Three-month-old 5xFAD mice were injected i.p. with PBS or type II T. gondii, and amyloid and IBA1+ cells were analyzed at 6 wpi. A) Representative images show amyloid (Amylo-Glo) signal in the subiculum of the hippocampus of PBS or T. gondii-injected mice. B) Average percent of amyloid area per FOV in each mouse. C) Average volume of amyloid plaque cores per FOV. D) Average numbers of amyloid plaques per FOV. E-F) Average percent of CD68 signal per FOV (E) and (F) CD68+amyloid+ signal per FOV in the. G-I) Average IBA1 (G), CD68 (H), and IBA1+CD68+ signal per FOV. J) IBA1 volume within a 15 µm radius of plaque cores was normalized to total plaque core volume per FOV in each animal. K-L) CD68 colocalization with amyloid (K) and in IBA1+ cells (L) per FOV in each animal. For B-L, n= 6-7 mice per group. Student’s t test, * p<0.05, **p<0.01, ns: not significant. Sup. Fig. 2: MAC2+ cells increase in the subiculum and some MAC2+ cells in the cortex contain amyloid. 5xFAD mice were injected with PBS or infected with T. gondii for 6 weeks, and markers for myeloid cell activation and amyloid were imaged in the subiculum of the hippocampus or cortex. A) Average percent of MAC2 volume per FOV in the subiculum per animal. B) Average percent of MAC2 and amyloid core colocalization per FOV in the subiculum per animal. n=7 mice per group. Student’s t test, **p<0.01, ***p<0.001. C) Representative FOV of amyloid colocalizing with CD68 (arrow head) in an IBA1+MAC2+ cell as seen in 3D view (top row) and inset orthogonal views (bottom rows). Sup. Fig. 3: Gating schema for infiltrating immune cells at 2, 4 and 6 wpi. 5xFAD and C57BL/6 mice were injected with PBS or infected with T. gondii, and brain homogenates were analyzed by flow cytometry. Representative gating scheme for quantifying lymphocytes (CD11b-CD45+), monocytes (CD45hiCD11b+), and microglia (CD11b+CD45int). Lympho [file 12974_2025_3666_MOESM2_ESM.pdf]

Yanes *et al.*
Supplemental Figures

Sup. Fig. 1

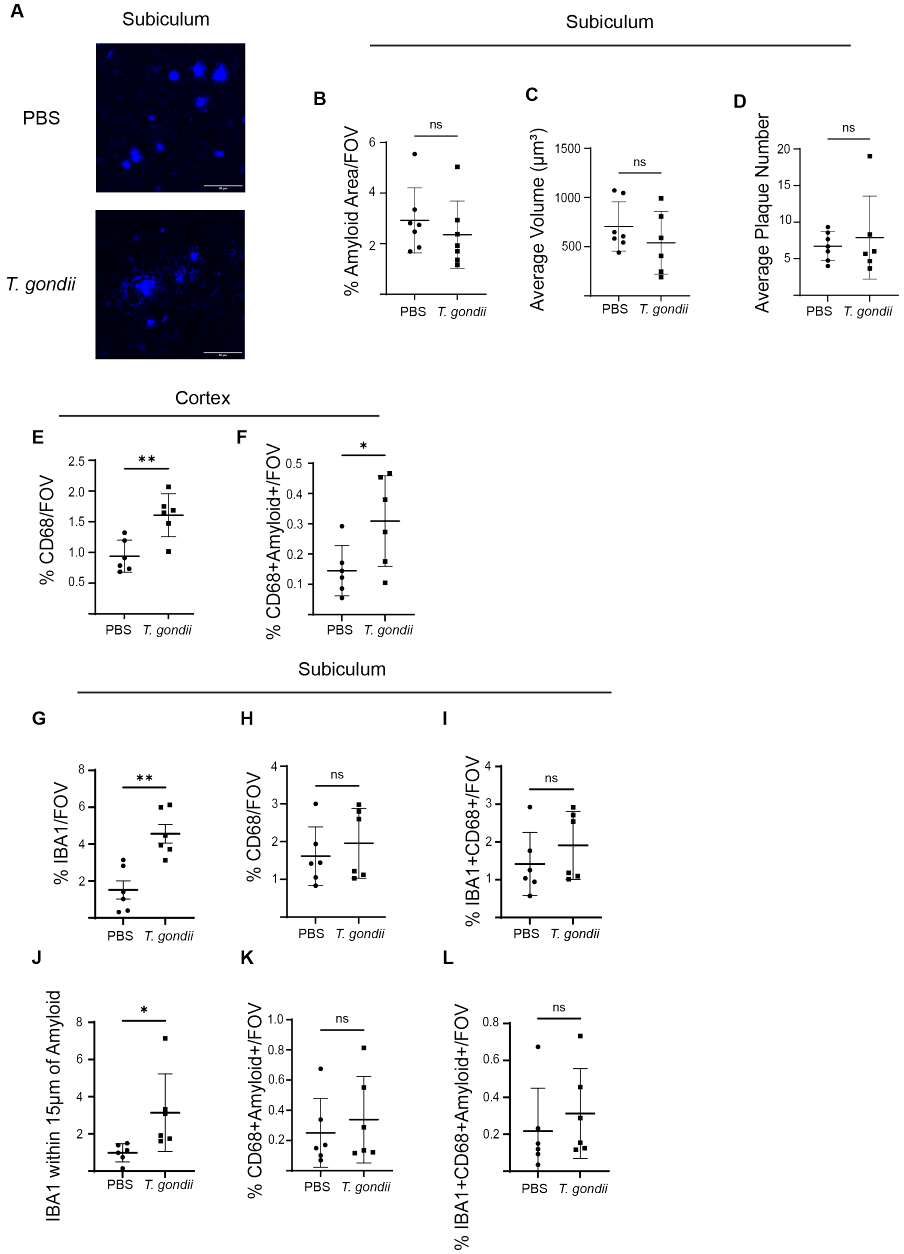

Sup. Fig 1: Amyloid and IBA1⁺ cells in the subiculum *T. gondii*-infected 5xFAD animals. Three-month-old 5xFAD mice were injected i.p. with PBS or type II *T. gondii*, and amyloid and IBA1$^+$ cells were analyzed at 6 wpi. **A)** Representative images show amyloid (Amylo-Glo) signal in the subiculum of the hippocampus of PBS or *T. gondii*-injected mice. **B)** Average percent of amyloid area per FOV in each mouse. **C)** Average volume of amyloid plaque cores per FOV. **D)** Average numbers of amyloid plaques per FOV. **E-F)** Average percent of CD68 signal per FOV (**E**) and (**F**) CD68$^+$amyloid$^+$ signal per FOV in the. **G-I)** Average IBA1 (**G**), CD68 (**H**), and IBA1$^+$CD68$^+$ signal per FOV. **J)** IBA1 volume within a 15 µm radius of plaque cores was normalized to total plaque core volume per FOV in each animal. **K-L)** CD68 colocalization with amyloid (**K**) and in IBA1$^+$ cells (**L**) per FOV in each animal. For **B-L**, n= 6-7 mice per group. Student's t test, * $p<0.05$, **$p<0.01$, ns: not significant.

Sup. Fig. 2

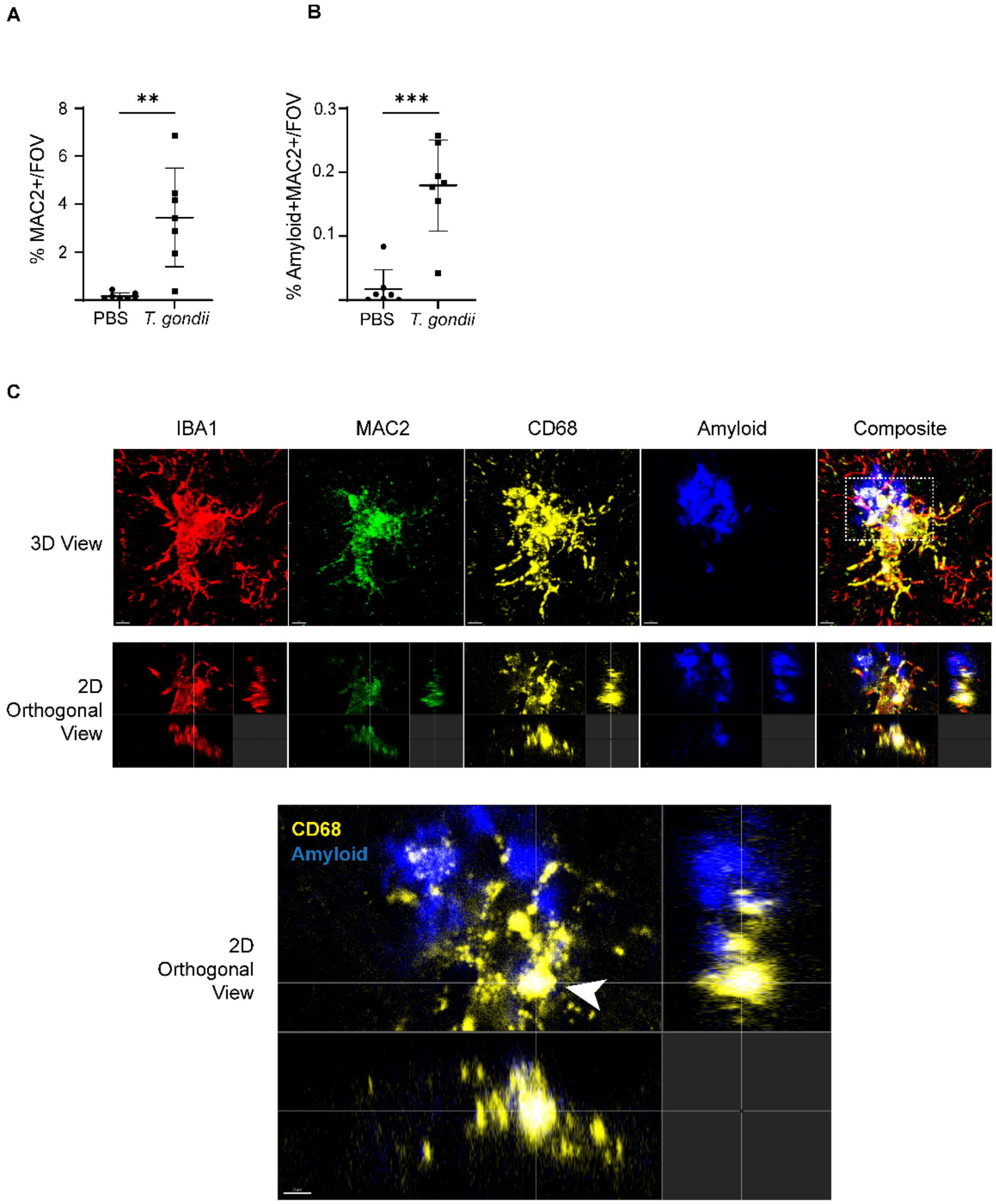

Sup. Fig. 2: MAC2$^+$ cells increase in the subiculum and some MAC2$^+$ cells in the cortex contain amyloid. 5xFAD mice were injected with PBS or infected with *T. gondii* for 6 weeks, and markers for myeloid cell activation and amyloid were imaged in the subiculum of the hippocampus or cortex. **A)** Average percent of MAC2 volume per FOV in the subiculum per animal. **B)** Average percent of MAC2 and amyloid core colocalization per FOV in the subiculum per animal. n=7 mice per group. Student's t test, **p<0.01, ***p<0.001. **C)** Representative FOV of amyloid colocalizing with CD68 (arrow head) in an IBA1$^+$MAC2$^+$ cell as seen in 3D view (top row) and inset orthogonal views (bottom rows).

Sup. Fig. 3

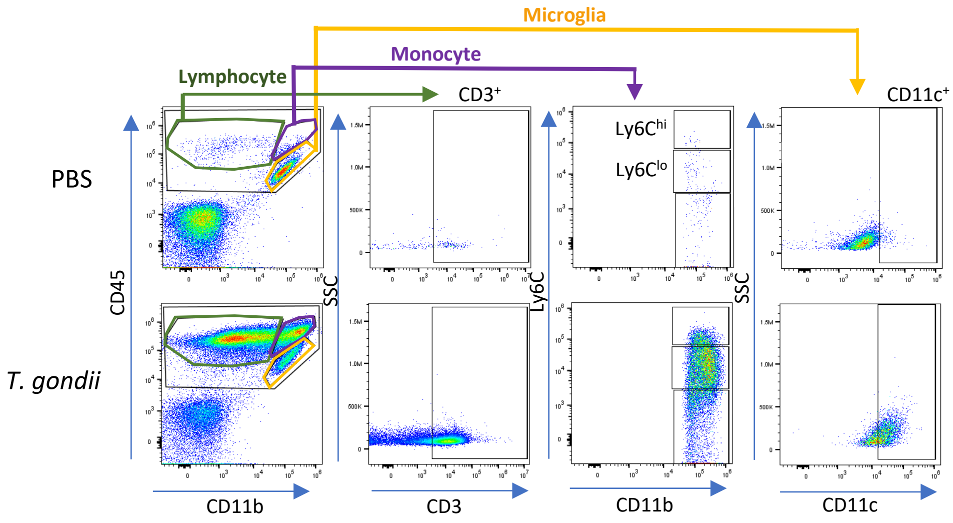

Sup. Fig. 3: Gating schema for infiltrating immune cells at 2, 4 and 6 wpi. 5xFAD and C57BL/6 mice were injected with PBS or infected with *T. gondii*, and brain homogenates were analyzed by flow cytometry. Representative gating scheme for quantifying lymphocytes ($CD11b^{-}CD45^{+}$), monocytes ($CD45^{hi}CD11b^{+}$), and microglia ($CD11b^{+}CD45^{int}$). Lymphocytes were further defined as $CD3^{+}$ T cells, monocytes as $Ly6C^{hi}$ or $Ly6C^{lo}$ monocytes, and microglia as $CD11c^{+}$.

Sup. Fig. 4

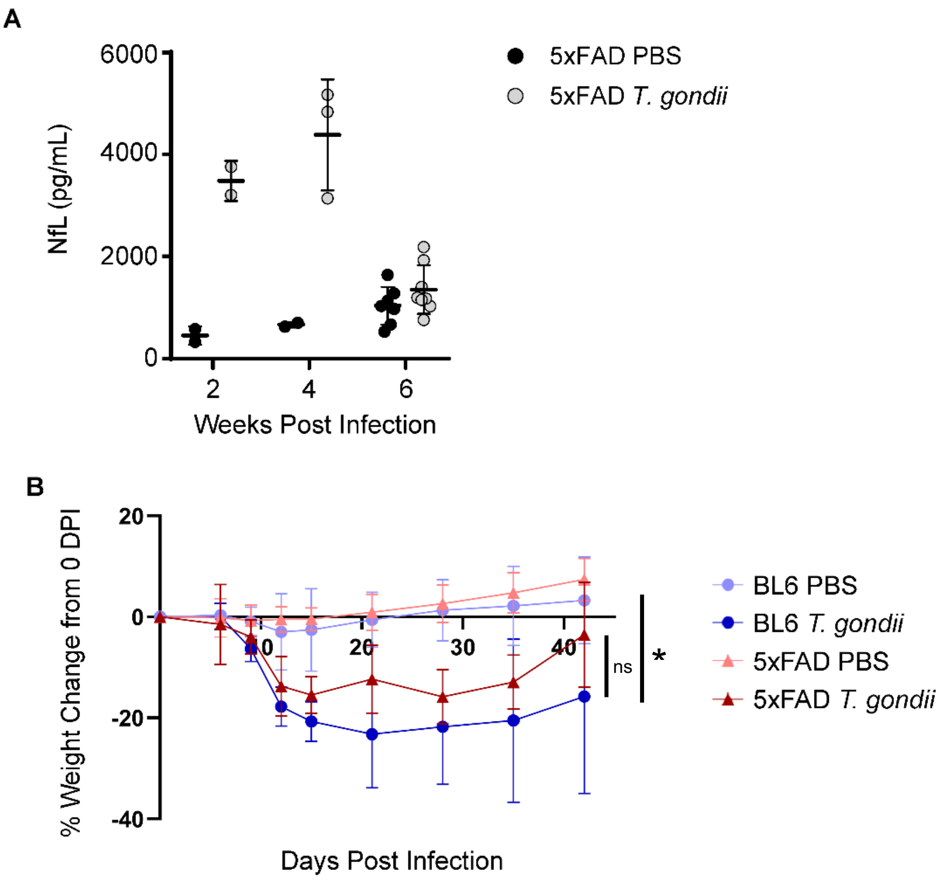

Sup. Fig. 4: Mice infected with *T. gondii* display systemic changes in neurofilament light and weight loss. **A)** 5xFAD mice were injected with PBS or infected with *T. gondii*, and levels of neurofilament light (NfL) were measured within plasma collected at 2, 4 and 6 weeks post-infection. n=2-8 per group **B)** Chimeric 5xFAD and C57BL/6 mice were injected with PBS or infected with *T. gondii* and weighed from 0-41 days post infection. % weight change from day 0 was calculated for each mouse and averaged across mice from days 2-6, 7-9, 10-12, 13-15, 16-21, 22-28, 29-35, 36-41. Endpoints compared using Student's t test. n=4-9 mice per group.

Sup. Fig. 5

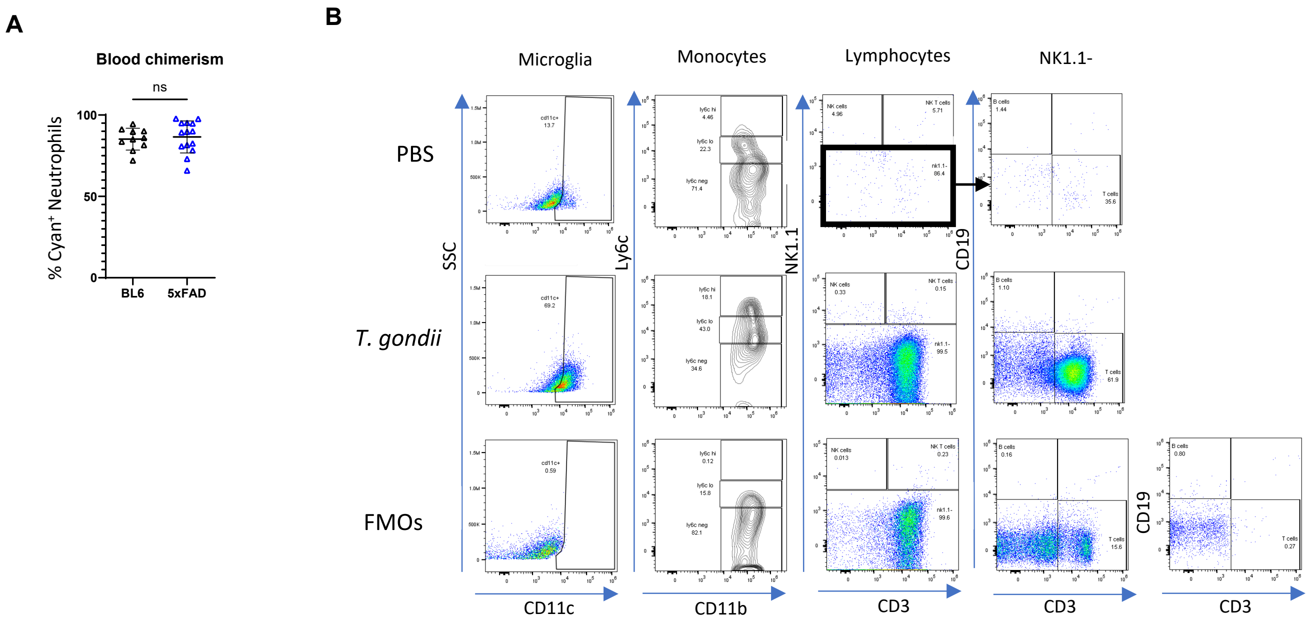

Sup. Fig. 5: Infiltrating immune cells are recruited to the chronically infected brain. 5xFAD and C57BL/6 mice were irradiated with a head shield and reconstituted with CAG-CFP bone marrow cells before infection with *T. gondii*. Six weeks later, brains were collected and processed for flow cytometry. **A)** Percent of cyan^{+} neutrophils in the peripheral blood of C57BL/6 (BL6) or 5xFAD mice at 11 weeks after bone marrow transplant as a measure of blood chimerism. **B)** Representative gating schema for cells from control and *T. gondii*-infected bone marrow chimeric mice.

Sup. Fig. 6

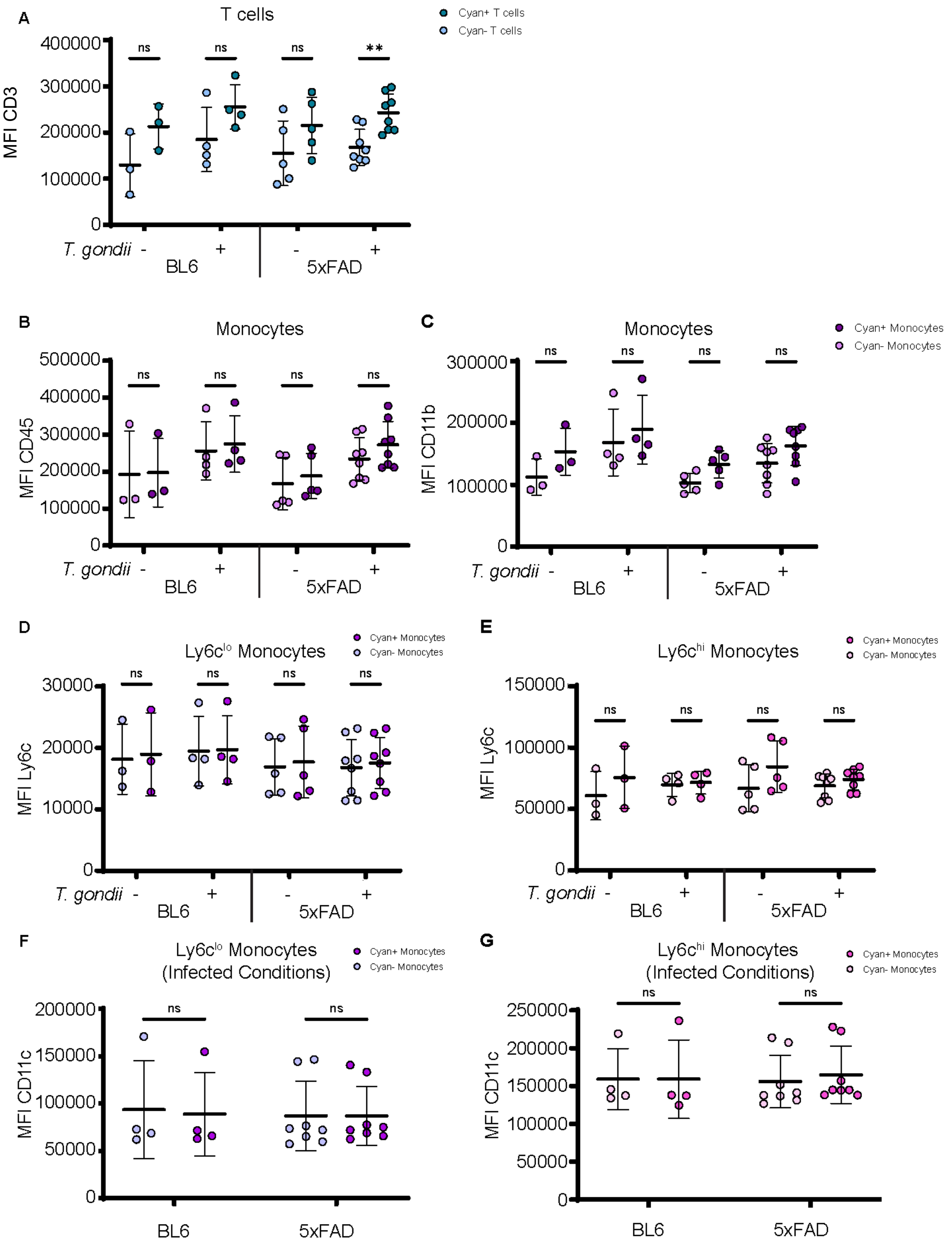

Sup. Fig. 6: Mean fluorescence intensity of phenotyping markers on cyan$^-$ and cyan$^+$ T cells and monocytes in the brain. 5xFAD and C57BL/6 mice were irradiated with a head shield and reconstituted with CAG-CFP (cyan) bone marrow cells before infection with *T. gondii*. Six weeks later, brains were collected and processed for flow cytometry. **A)** CD3 expression as measured by geometric mean fluorescence intensity (MFI) for cyan$^+$ and cyan$^-$ CD3$^+$ T cells. **(B)** CD45 and **(C)** CD11b MFI expression in CD11b$^+$CD45^{hi} monocytes. Ly6c MFI expression in **(D)** CD11b$^+$CD45^{hi}Ly6c^{lo} monocytes and **(E)** CD11b$^+$CD45^{hi}Ly6c^{hi} monocytes. CD11c MFI expression in **(F)** CD11b$^+$CD45^{hi}Ly6c^{lo} monocytes and **(G)** CD11b$^+$CD45^{hi}Ly6c^{hi} monocytes. In **A-G,** each dot represents one animal. n=3-8 mice per group. Two-way ANOVA followed by a post-hoc Fisher's LSD, **p<0.01, ns: not significant.

Sup. Fig. 7

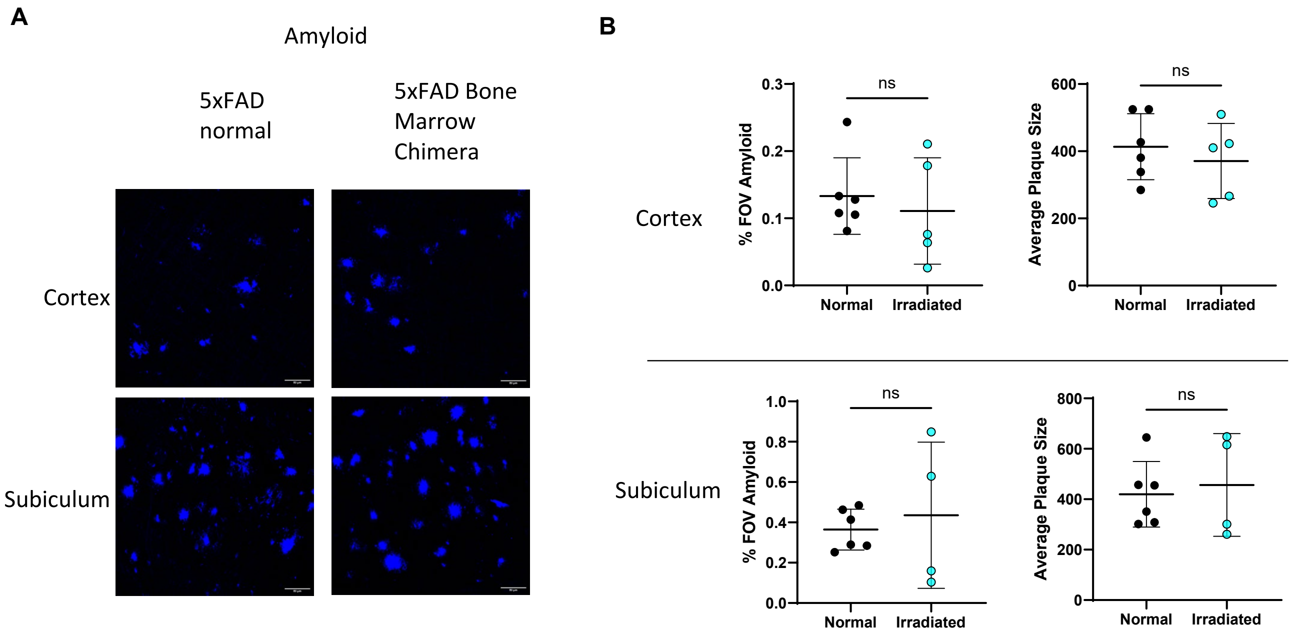

Sup. Fig. 7: Amyloid burden in uninfected 18-week-old 5xFAD mice with and without bone marrow transplantation. 5xFAD (unirradiated, untransplanted) and chimeric 5xFAD (irradiated and transplanted with CAG-CFP bone marrow) mice were injected with PBS at 12 weeks of age and analyzed 6 weeks later. **A)** Representative images from the cortex and subiculum of mice without (left) and with (right) irradiation and peripheral bone marrow reconstitution. **B)** The percent area of amyloid per FOV and the average plaque size in control and irradiated mice. In **A** and **B,** data reflect averages of 1-4 FOVs per section, 2 sections per animal. $n_{5xFAD\ normal}$ = 6, $n_{5xFAD\ irradiated}$ = 4. Student's t test, ns: not significant.

Sup. Movie 1

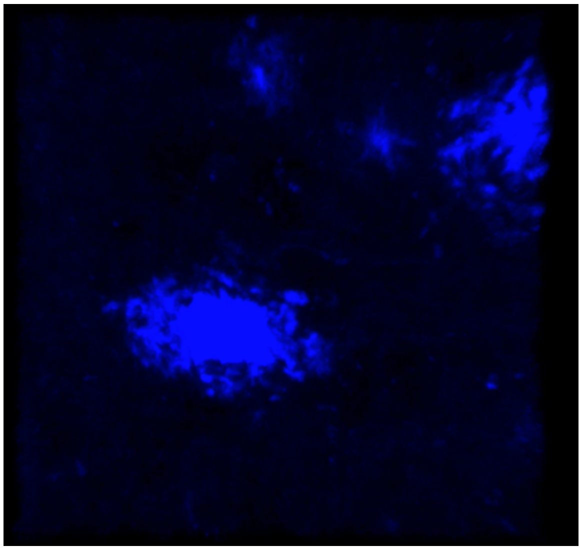

Movie S1. **Amyloid signal is detectable inside IBA1$^+$cyan$^+$ cells**. This movie represents the inset image from Fig. 7A. Chimeric 5xFAD mice were injected with *T. gondii*, and 6 weeks later the brain sections were imaged using confocal microscopy for amyloid (Amylo-Glo), IBA1, and cyan$^+$ cells. Surfaces were generated for each channel in Imaris, and amyloid signal (blue) is within a cell that is both IBA1$^+$ (red) and peripherally derived (cyan$^+$).
